# Ddx20, an Olig2 binding factor, governs the survival of neural and oligodendrocyte progenitor cells via proper Mdm2 splicing and p53 suppression

**DOI:** 10.1038/s41418-021-00915-8

**Published:** 2022-01-01

**Authors:** Norihisa Bizen, Asim K. Bepari, Li Zhou, Manabu Abe, Kenji Sakimura, Katsuhiko Ono, Hirohide Takebayashi

**Affiliations:** 1 Division of Neurobiology and Anatomy, Graduate School of Medical and Dental Sciences, Niigata University, Niigata, Japan; 2 Department of Pharmaceutical Sciences, North South University, Dhaka, Bangladesh; 3 Department of Cellular Neurobiology, Brain Research Institute, Niigata University, Niigata, Japan; 4 Center for Coordination of Research Facilities (CCRF), Niigata University, Niigata, Japan; 5 Department of Animal Model Development, Brain Research Institute, Niigata University, Niigata, Japan; 6 Developmental Neurobiology, Kyoto Prefectural University of Medicine, Kyoto, Japan

**Keywords:** Oligodendrocyte, Experimental models of disease

## Abstract

Olig2 is indispensable for motoneuron and oligodendrocyte fate-specification in the pMN domain of embryonic spinal cords, and also involved in the proliferation and differentiation of several cell types in the nervous system, including neural progenitor cells (NPCs) and oligodendrocytes. However, how Olig2 controls these diverse biological processes remains unclear. Here, we demonstrated that a novel Olig2-binding protein, DEAD-box helicase 20 (Ddx20), is indispensable for the survival of NPCs and oligodendrocyte progenitor cells (OPCs). A central nervous system (CNS)-specific *Ddx20* conditional knockout (cKO) demonstrated apoptosis and cell cycle arrest in NPCs and OPCs, through the potentiation of the p53 pathway in DNA damage-dependent and independent manners, including SMN complex disruption and the abnormal splicing of *Mdm2* mRNA. Analyzes of *Olig2* null NPCs showed that Olig2 contributed to NPC proliferation through Ddx20 protein stabilization. Our findings provide novel mechanisms underlying the Olig2-mediated proliferation of NPCs, via the Ddx20-p53 axis, in the embryonic CNS.

## Introduction

During neural development, neuroepithelial cell fate is determined by region-specific transcription factors, the expression of which is regulated by morphogens, resulting in various types of neurons and glial cells being generated in a region-specific manner [1]. Oligodendrocyte transcription factor 2 (Olig2) is a basic helix-loop-helix transcription factor, involved in the dorsoventral patterning of embryonic spinal cords, and is indispensable for the fate specification of several neurons and glial cells in the central nervous system (CNS). Olig2 is expressed in the ventral ventricular zone (VZ), termed pMN domain, where oligodendrocyte progenitor cells (OPCs) are produced. Several studies, both in vivo and in vitro, have demonstrated that Olig2 regulates not only motor neurons and OPC production from the pMN domain [2–4] but also the neural progenitor cell (NPC) proliferation [5] and oligodendrocyte differentiation from OPCs [6]. Furthermore, Olig2 has been implicated in the production of astrocyte subpopulations [7] and cholinergic neurons [8] in the developing CNS and the proliferation of glioma stem cells [5]. However, how Olig2 controls these diverse biological processes remains largely unknown.

In this study, using yeast two-hybrid screening, we identified the DEAD (Asp-Glu-Ala-Asp)-box protein Ddx20 (also known as Gemin3 or DP103), as a novel Olig2-interacting factor. Ddx20 engages in various cellular processes, such as transcription, RNA splicing, and translation, as described below. 1) Ddx20 binds to some transcription factors and transcription regulatory factors, to control the transcription of targeted genes [9]. 2) Ddx20 directly interacts with survival of motor neurons (SMN) protein, and the SMN complex contributes to the regulation of splicing, via the assembly of spliceosomal small nuclear ribonucleoproteins (snRNPs) [10]. 3) Ddx20 interacts with Argonaute 2 (Ago2, also known as Eif2c2) and complexes with RNA-inducing silencing complex to promote the biogenesis of microRNAs (miRNAs) [11], which mediate translational inhibition and RNA degradation. There are reports on loss of function of *Ddx20* in *Drosophila* and *C. elegans*, which leads to the loss of viability, dysfunction of neuromuscular junction, and abnormal motor behavior [12, 13]. However, the in vivo functions of Ddx20 in mammals including mice remain poorly understood, because conventional *Ddx20* knockout (KO) mice are lethal by the four-cell stage [14].

We thus produced CNS-specific *Ddx20* conditional KO (cKO) mice to analyze the function of Ddx20 during neural development, demonstrating that Ddx20 is indispensable for the survival of NPCs and OPCs. We further show that Ddx20 contributes to SMN stabilization and suppresses the p53 pathway through genome stabilization and appropriate control of *Mdm2* splicing. Notably, Olig2 contributes to NPC proliferation by suppressing the p53 pathway through the stabilization of Ddx20 protein. Taken together, our findings uncovered a novel molecular mechanism for NPC and OPC maintenance, which is indispensable for normal neural development.

## Materials and methods

### Animals

Mice carrying floxed *Ddx20* alleles were generated as described in Supplemental Materials and Methods. Following mice lines were used in the study: *Nestin*-*Cre* transgenic mice [15] (MGI:2176173), *Cnp-iCre* knockin mice in which *iCre* cassette was inserted into exon 1 of *Cnp* locus (Supplemental Fig. 5), *Olig2-CreER* mice (RBRC01507, MGI:2183410) [2], *Z/EG* reporter mice (MGI:3046177) [16], *p53* knockout mice (RBRC01361, MGI:1926340) [17], which were obtained from RIKEN BRC. Genotyping was performed as previously described [15–17]. For the genotyping of *Cnp-iCre* knockin mice, PCR consisted of 30 cycles of denaturation at 94 ˚C for 30 s, annealing at 60˚C for 30 s, and extension at 68˚C for 60 s. PCR primers for genotyping were listed in Supplementary Table S1. NPC- or OPC-specific *Ddx20* cKO mice were generated by crossing *Nestin*-*Cre* transgenic male mice or *Cnp-iCre* knockin male mice, respectively. For the lineage trace of Olig2 expressing cells in *Ddx20* deficient mice, *Ddx20*^*flox/flox*^ female mice were crossed with *Ddx20*^*+/−*^;*Olig2-CreER;*Z/EG male mice. To obtain the NPC-specific *Ddx20* and *p53* double deficient mice, *Nestin-Cre*;*Ddx20*^*flox/+*^; *p53*^*+/−*^ male mice were crossed with *Ddx20*^*flox/flox*^; *p53*^*+/−*^ female mice, resulting in the generation of *Nestin-Cre*;*Ddx20*^*flox/flox*^; *p53*^*−/−*^ mice. Mice are maintained on a 12-h light/dark cycle with ad libitum access to food and water. For NSC preparation, pregnant ICR mice were purchased from Japan SLC, Inc (Shizuoka, Japan). All animal experiments were conducted in accordance with the guidelines of Niigata University Animal Care and Use Committee. The gender of all mouse embryos was not determined.

### Cell lines

HEK293 cells (ATCC, #CRL-1573) were used in this study. Plate-E cells were established and kindly provided by T. Kitamura’s laboratory (University of Tokyo). The cell lines were tested for mycoplasma contamination.

### Immunohistochemistry

Immunohistochemistry was performed as previously described [18] with minor modification. Detailed procedures of histological analyses are described in the Supplementary Materials and Methods. Primary antibodies were used bellow: rabbit anti-Ddx20 (1:200, ABclonal, Cat#A5817), mouse anti-Olig2 (1:500, Millipore, Cat#MABN50), mouse anti-CC1 (1:200, Novus Biologicals, Cat#NB600-1021), Biotin-conjugated rat anti-PDGFRα (1:200, eBioscience, Cat#13-1401-82), mouse anti-Nestin (1:200, DSHB, Cat#Rat-401), rabbit anti-Sox2 (1:500, Abcam, Cat#ab97959), mouse anti-βIII-Tubulin (1:1,000, BioLegend, Cat#MMS-435P), rabbit anti-GFAP (1:1,000, Dako, Cat#Z0334), rat anti-Glutamine Synthetase (GS) (1:300, BD Biosciences, Cat#610517), mouse anti-ALDH1L1 (1:200, NeuroMab, Cat#73-140), rabbit anti-cleaved Caspase-3 (1:800, Cell Signaling Technology, Cat#9664), rabbit anti-γH2AX (1:200, Active Motif, Cat#39117), rabbit anti-p53 (1:200, Leica Biosystems, Cat# NCL-L-p53-CM5p), rat anti-BrdU (1:300, Abcam, Cat#ab6326), mouse anti-Hb9 (1:50, DSHB, Cat#81.5C10), mouse anti-Islet1 (1:100, DSHB, Cat#39.4D5), rabbit anti-GFP (1:1000, MBL, Cat#598), rat anti-GFP (1:300, nacalai tesque, #04404-84). Appropriate secondary antibodies conjugated to Alexa Fluor dye (1:1,000, Thermo Fisher Scientific) or Avidin conjugated to Alexa Fluor dye (1:1000, Thermo Fisher Scientific) were used.

### In situ hybridization (ISH)

ISH was performed as previously described with minor modifications [18]. A detailed description is in the Supplementary Materials and Methods.

### Co-immunoprecipitation (Co-IP) and western blotting

Co-IP and western blotting were performed as previously described [19] with minor modifications (Supplementary Materials and Methods). In Fig. 6A, B, D–G, M, N, the intensity of each lane was measured by ImageJ software (https://imagej.nih.gov/ij/). Following antibodies were used for Co-IP: rabbit anti-Ddx20 antibody (10 µg, homemade, Immunogen is 722-740 aa of mouse Ddx20), mouse anti-FLAG M2 (1:1,000, mouse, Sigma, F1804), normal rabbit IgG (10 µg, MBL, Cat#PM035). Following antibodies were used for western blotting: rabbit anti-HA (1:1,000, MBL, Cat#561), mouse anti-FLAG M2 (1:1,000, mouse, Sigma, F1804), mouse anti-Myc (1:1000, DSHB, 9E10), rabbit anti-Ddx20 antibody (1:1,000, homemade), mouse anti-Olig2 antibody (1:1,000, Millipore, Cat#MABN50), mouse anti-SMN (1:1000, BD Biosciences, Cat#610646), mouse anti-Gemin2 (1:1000, BioLegend, Cat#862902), mouse anti-Gemin6 (1:1000, BioLegend, #862302), mouse anti-β-actin (1:2,000, Sigma-Aldrich, Cat#AC-15), mouse anti-α-Tubulin (1:2000, Cell Signaling Technology, Cat#3873), horseradish peroxidase (HRP)-labeled anti-rabbit IgG, HRP-labeled anti-mouse IgG (1:2,000, Cell Signaling Technology, Cat#7074, 7076).

### NPC culture and neurosphere assay

NPC culture was performed as previously described [19] with minor modifications. A detailed description is in the Supplementary Materials and Methods.

### Splice-switching in *Mdm2* mRNA in vivo

Vivo morpholino (MO) targeting the 5′ splice sites of *Mdm2* exon3 was purchased from Gene tools [20], and the sequences are listed in Supplementary Table S1. 1 µl of 1 µg/µl MO mixed with saline containing 0.1% Fast Green was injected into the ventricles of the embryonic mouse brains at E14.5. Nighty-six hours after injection, the brains were collected and used for immunohistochemistry or RT-qPCR.

### Cycloheximide chase assay

HA-tagged Ddx20 expression plasmids were cotransfected with or without Myc-Olig2 expression plasmids into Plat-E cells. The cells were treated with cycloheximide (CHX; 200 μg/ml, Wako) for 6, 18, and 24 h. A detailed description is in the Supplementary Materials and Methods.

### Retrovirus preparation

Retrovirus was prepared as previously described with minor modifications [19]. A detailed description is in the Supplementary Materials and Methods.

### Quantification of p53 intensity in nuclei of NPCs

The mean density of p53 signals in the nuclei of GFP-positive cells was quantified by converting to the number of pixels using ImageJ in Fig. 6H. The mean density of p53 signals was determined by measuring the integrated density of p53 in the nuclei of GFP-positive cells and dividing that value by the area of the DAPI-labeled nuclei.

### Reverse transcription PCR (RT-PCR) and RT-quantitative PCR (RT-qPCR)

RT-PCR and RT-qPCR were performed as described previously [21] with minor modifications. A detailed description is in the Supplementary Materials and Methods. Primers used for PCR were described in Supplementary Table S1.

### RNA sequence analysis and data analysis

Total RNA extraction, preparation of mRNA libraries, and sequencing were performed as previously described with minor modification [22]. Briefly, total RNA was extracted from E14.5 mouse spinal cords of two independent *Ddx20*^*flox/flox*^ and *Nestin-Cre;Ddx20*^*flox/flox*^ littermates using miRNeasy mini kit (Qiagen). The integrity and quantities of extracted RNA were assessed using an Agilent 2100 Bioanalyzer (Agilent Technologies), followed by the preparation of mRNA libraries using Illumina TruSeq protocols for polyA selection, fragmentation, and adapter ligation (TruSeq RNA sample preparation kit version 2, Illumina). The multiplexed libraries were sequenced as 150 nt paired-end runs on Illumina HiSeq 4000 at Novogene company (https://en.novogene.com/). Sequence reads were aligned to the reference mouse genome (mm10) using OLego [23], and expression levels and alternative splicing events were quantified by Quantas [24] (https://zhanglab.c2b2.columbia.edu/index.php/Quantas). Differential expression analysis of mRNA abundance was performed by edgeR [24]. The calculation of the exon inclusion rate and differential exon inclusion rate of each mRNA between two groups (Δ*I*) were statistically analyzed by Fisher exact test in the Quantas tool. The statistical criteria (FDR < 0.1 and *p* < 0.001) as significant changes of expression and alternative splicing was employed. Volcano plots and heatmaps were drawn using ggplot2 and heatmap.2 in R (version 3.6.3), respectively.

### Gene Ontology (GO) analysis

GO analysis of either differential expression or altered splicing was performed by Metascape (https://metascape.org). The set of genes whose either expression or alternative splicing was significantly altered was subjected to GO analysis. GO terms with p-values less than 0.05 were enriched and listed in order of significance. Among them, Fig. 4B showed the top 5 GO terms with the biological process in the list of significant down- or up-regulated genes, respectively.

### Statistical analyses

All experiments were performed at least three biological replicates unless otherwise stated, and the values were presented as means ± SD, or means ± SEM (Fig. 4J). In animal experiments, the gender of mouse embryos was not determined and no blinding was performed. Statistical analysis was performed using Excel 2013 or R (version 3.6.3). Statistical significance was determined by a two-tailed unpaired *t*-test in almost all data. For groups with equal variance, Student’s *t*-test was performed, otherwise, Welch’s *t* test was performed. One-way analysis of variance with post hoc Tukey’s test was performed in Fig. 4J. Kruskal-Wallis test with post hoc Steel-Dwass test was performed in Fig. 6G, H and Supplementary Fig. S10B. The *p*-value of <0.05 was considered to be statistically significant.

## Results

### Identification of Ddx20 as a novel Olig2-binding factor

**Fig. 1:**
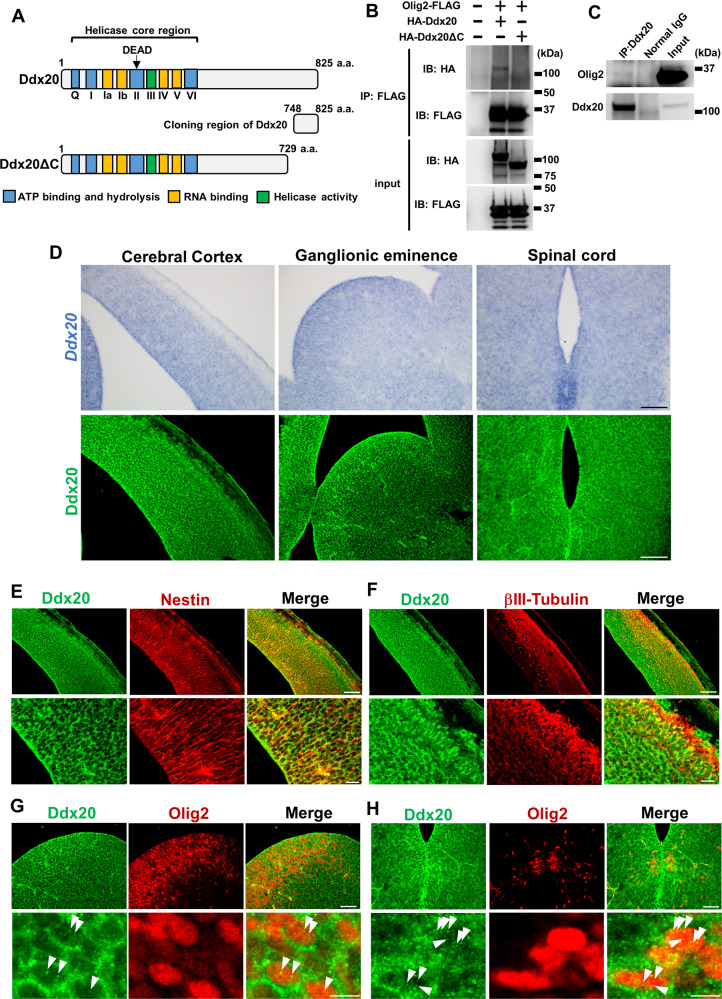
Identification of a novel Olig2-binding protein, Ddx20. **A** A schematic showing the structure of Ddx20. Nine motifs can be found in the N-terminus of Ddx20 (Q, I, Ia, Ib, II, III, IV, V, VI), which are conserved across the DEAD-box protein family, as a helicase core. The functions of each motif are indicated. The C-terminus contains binding motifs for some transcription factors. Yeast two-hybrid screening identified the C-terminal fragment of Ddx20 (748–825 aa) as the Olig2-binding region. **B** FLAG-tagged Olig2 co-IP analysis in HEK293 cells transfected with HA-tagged Ddx20 and HA-tagged Ddx20ΔC, a truncated form of Ddx20 lacking the C-terminal region (730–825 aa). Immunoprecipitates and input samples were analyzed with anti-HA and anti-FLAG antibodies. A representative result from two independent experiments is shown. **C** Endogenous interaction between Olig2 and Ddx20 was detected in cultured NPCs, derived from mouse embryonic telencephalons, at E14.5. A representative result from two independent experiments is shown. **D** In situ hybridization and immunohistochemistry demonstrating *Ddx20* mRNA and Ddx20 protein expression, respectively, in mouse embryonic cerebral cortex, ganglionic eminence, and spinal cord, at E13.5. Scale bars, 100 μm. **E**, **F** Double-immunostaining against Ddx20 and Nestin or Ddx20 and βIII-Tubulin, in mouse cerebral cortex sections, at E13.5. Bottom images show high-magnification images. Scale bars, 100 μm (upper images); 20 μm (bottom images). Double immunohistochemistry staining against Ddx20 and Olig2, in the lateral ganglionic eminence (**G**) and spinal cord (**H**), at E13.5. Bottom images show high-magnification images. White arrowheads indicate Ddx20 puncta in the nucleus of representative Olig2-expressing cells. Scale bars, 50 μm (upper images); 10 μm (bottom images).

To identify novel factors that interact with Olig2 during neural development, we screened novel Olig2-binding proteins, by performing a yeast two-hybrid screening, using full-length Olig2 as bait. A mouse embryonic brain cDNA library, fused with the LexA activation domain, was screened, resulting in the identification of a cDNA fragment encoding the C-terminal region (748–825 aa) of Ddx20, which is also known as Gemin3 or DP103 (Fig. 1A). Co-immunoprecipitation analysis, using tagged proteins expressed in HEK293 cells, demonstrated an interaction between Olig2 and Ddx20, but no interaction was observed between Olig2 and a C-terminal-truncated form of Ddx20 (Fig. 1B). The interaction between endogenous Olig2 and Ddx20 was confirmed in cultured NPCs, derived from embryonic mouse telencephalons (Fig. 1C). We next investigated Ddx20 expression patterns in the embryonic CNS. In situ hybridization and immunohistochemistry analyses demonstrated that Ddx20 was widely expressed in the embryonic brain and spinal cord, at E13.5; however, Ddx20 was strongly expressed in the VZ and the subventricular zone (SVZ), where NPCs exist (Fig. 1D). Ddx20 was expressed in NPCs (Fig. 1E), neurons (Fig. 1F), and Olig2-positive cells in the ganglionic eminence and spinal cord (Fig. 1G, H). Ddx20 was diffusely distributed throughout the cytoplasm and localized in discrete nuclear foci, called gems [25]. These puncta were also found in the nuclei of Olig2-positive cells (Fig. 1G, H).

### Appearance of neural progenitor cell apoptosis in CNS-specific *Ddx20* cKO forebrains

**Fig. 2:**
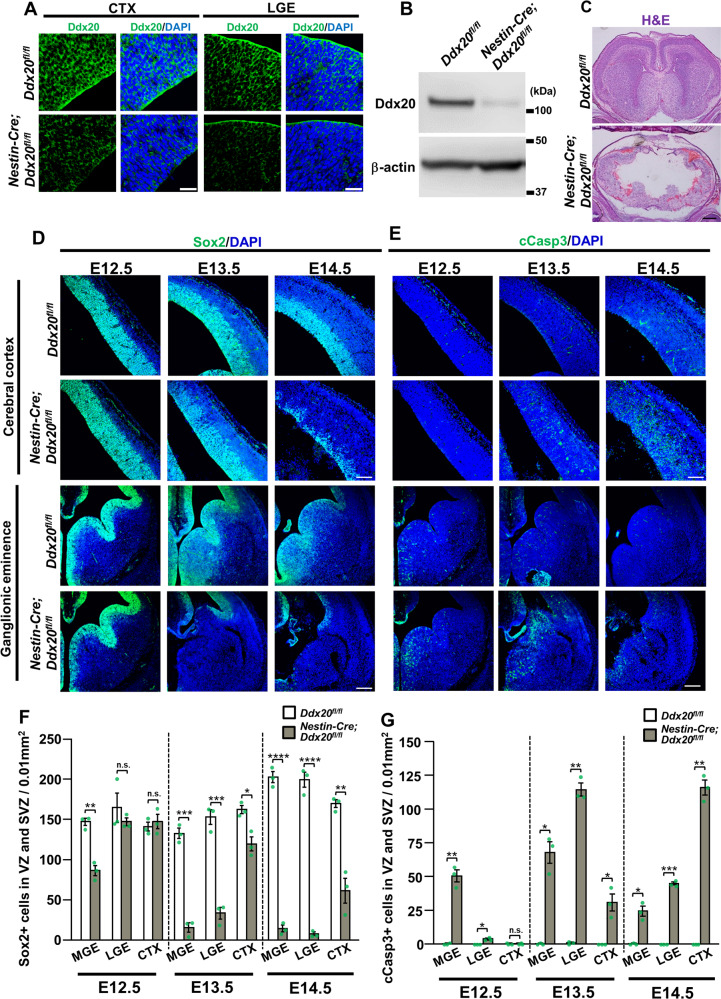
Progressive cell death of neural progenitor cells in CNS-specific *Ddx20* cKO forebrains. **A** Validation of Ddx20 downregulation in *Nestin-Cre;Ddx20* cKO brains, at E13.5, using immunohistochemistry. CTX; cerebral cortex, LGE; lateral ganglionic eminence. **B** Immunoblotting for Ddx20 and β-actin, in control and *Nestin-Cre;Ddx20* cKO brains, at E13.5. **C** Hematoxylin and eosin (H&E) staining to examine the morphological phenotypes of control and *Nestin-Cre;Ddx20* cKO brains, at E17.5. **D**, **E** Immunostaining for Sox2 and cleaved caspase-3 (cCasp3), at E12.5, E13.5, and E14.5, in the forebrains of control and *Nestin-Cre;Ddx20* cKO mice. **F**, **G** Quantitative data showing the numbers of Sox2- or cCasp3-positive cells per 0.01 (mm^2^), in the VZ/SVZ of the embryonic forebrain, (shown in **D** and **E**). *n* = 3 mice per group. Bar charts represent the mean ± SD. Statistical analysis was performed by two-tailed unpaired *t*-test. *p < 0.05; **p < 0.01; ***p < 0.001; ****p < 0.0001; n.s., not significant. Scale bars, 30 μm (**A**); 400 μm (**C**); 100 μm (**D** and **E** CTX); 200 μm (**D** and **E** LGE).

To elucidate the role played by Ddx20 during CNS development, we generated CNS-specific *Ddx20* cKO mice, using a *Nestin-Cre* driver (Supplementary Fig. S1A–C). We confirmed the decreased expression of Ddx20 proteins in the embryonic forebrains of *Ddx20* cKO mice (*Nestin-Cre*;Ddx20^*flox/flox*^), at E13.5 (Fig. 2A, B). *Ddx20* cKO mice died immediately after birth, with severe disruptions in the brain structures (Fig. 2C and Supplementary Fig. S1D, E). Sox2-targeted immunohistochemistry demonstrated the severe loss of NPCs in the VZ/SVZ. Defects in Sox2-positive NPCs progressed, from the pre-optic area and the medial ganglionic eminence (MGE) to the lateral ganglionic eminence (LGE) (Fig. 2D). Moreover, in the cerebral cortex, significant defects were observed, starting from E14.5 (Fig. 2D, F). Next, we performed cleaved Caspase-3 (cCasp3) staining, to determine whether the observed NPC loss was caused by apoptosis. The appearance of cCasp3-positive cells was well-correlated with the loss of Sox2-positive cells (Fig. 2E). In the basal ganglia, cCasp3-positive cells first appeared in the pre-optic area and then shifted to the MGE/LGE. In the cerebral cortex, cCasp3-positive cells began to appear at E13.5 and nearly covered the entire cortex by E14.5 (Fig. 2E, G). From these data, Ddx20 appears to be essential for the survival of NPCs in embryonic forebrains.

### Severe defects were observed in oligodendrocyte progenitor cells in the spinal cords of CNS-specific *Ddx20* cKO mice

**Fig. 3:**
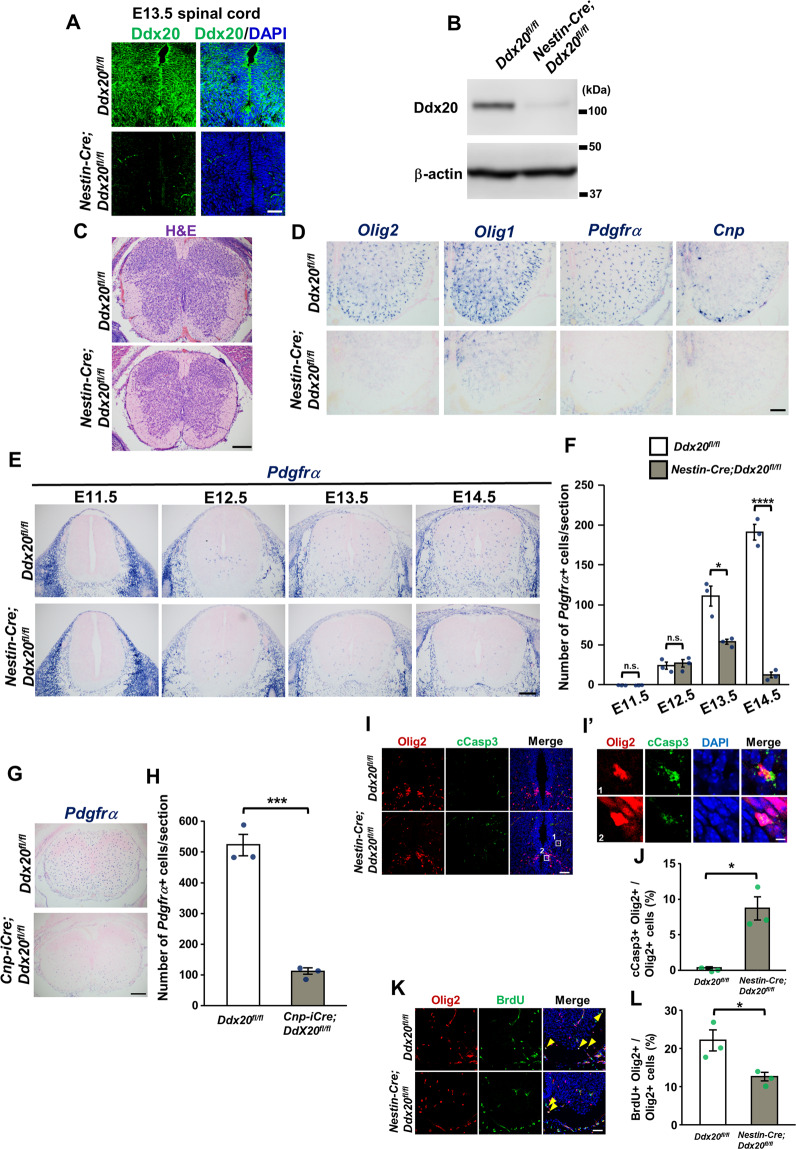
*Ddx20* ablation induced apoptosis and cell cycle arrest in OPCs from embryonic spinal cords. **A** Immunohistochemistry for Ddx20, in control and *Nestin-Cre;Ddx20* cKO spinal cords, at E13.5. **B** Western blotting for Ddx20 and β-actin, in control and *Nestin-Cre;Ddx20* cKO spinal cords, at E13.5. **C** H&E staining, in control and *Nestin-Cre;Ddx20* cKO spinal cords, at E17.5. **D** In situ hybridization for oligodendrocyte-related genes (*Olig2*, *Olig1*, *Pdgfrα*, and *Cnp*), in control and *Nestin-Cre;Ddx20* cKO mice. **E** Comparison of *Pdgfrα* gene expression between control and *Nestin-Cre;Ddx20* cKO spinal cords, from E11.5 to E14.5. **F** The average number of *Pdgfrα*-positive cells per section, shown in **E**. *n* = 3 mice per group. **G** In situ hybridization for *Pdgfrα*, in control and *Cnp-iCre;Ddx20* cKO spinal cords, at E17.5. **H** The average number of *Pdgfrα*-positive cells per section, shown in **G**. *n* = 3 mice per group. **I** Immunohistochemistry for cCasp3 and Olig2, in control and *Nestin-Cre;Ddx20* cKO spinal cords, at E14. **I’** High-magnification and representative images of cCasp3 and Olig2 double-positive cells, from **I**. **J** The ratio Olig2 and cCasp3 double-positive cells to Olig2 single-positive cells, shown in **I**. *n* = 3 mice per group. **K** Immunostaining for BrdU and Olig2, in control and *Nestin-Cre;Ddx20* cKO spinal cords, at E13.5. **L** The ratio Olig2 and BrdU double-positive cells to Olig2 single-positive cells, shown in **K**. *n* = 3 mice per group. Bar charts represent the mean ± SD. Statistical analysis was performed by two-tailed, unpaired *t*-test. *p < 0.05; ***p < 0.001; ****p < 0.0001; n.s., not significant. Scale bars, 50 μm (**A**, **I**, and **K**); 200 μm (**C**, **E**, and **G**); 100 μm (**D**); 5 μm (**I’**).

The decreased expression of Ddx20 protein was also confirmed in CNS-specific *Ddx20* cKO spinal cords (Fig. 3A, B); however, no apparent disruption in the VZ structure or disappearance of Sox2-positive NPCs was observed during the embryonic period (Fig. 3C and Supplementary Fig. S2A). In contrast, marked defects in oligodendrocytes were observed in the spinal cord, at E17.5 (Fig. 3D). Although the loss of Ddx20 did not affect the number of motor neurons (Supplementary Fig. S2B, C), astrocyte differentiation tended to be suppressed (Supplementary Fig. S3A, B). To investigate the influence of *Ddx20* deficiency on oligodendrogenesis in Olig2-expressing cells in the pMN domain, we generated a conditional *Ddx20* deletion in Olig2-positive cells, by crossing *Ddx20*^*floxl/flox*^ mice with mice harboring the *Olig2-CreER* knock-in allele [2] and the *lacZ/EGFP* (*Z/EG*) reporter allele. Tamoxifen was intraperitoneally injected into pregnant mice, at E10.5, and the embryos were analyzed at E18.5 (Supplementary Fig. S4A, B). The ratios of Olig2- or CC-1-positive cells to green fluorescent protein (GFP)-positive cells in *Ddx20* cKO spinal cords significantly decreased compared with those of control spinal cords (Supplementary Fig. S4C–F). Next, we found a significant loss in platelet-derived growth factor receptor α (*Pdgfrα*)-positive OPCs, starting at E13.5 but not E12.5, when oligodendrogenesis begins, indicating that *Ddx20* deficiency impairs OPC maintenance but not OPC production (Fig. 3E, F). To further investigate whether the loss of OPCs was due to the cell-autonomous effects, we analyzed the spinal cords of OPC-specific *Ddx20* cKO mice (*Cnp-iCre;Ddx20*^*flox/flox*^) (Supplementary Fig. S5). The number of *Pdgfrα*-positive OPCs in OPC-specific *Ddx20* cKO spinal cords was significantly lower than that in control spinal cords (Fig. 3G, H), suggesting cell-autonomous effects for Ddx20 in OPCs. Unlike the CNS-specific *Ddx20* cKO mice, OPC-specific *Ddx20* cKO mice did not show the apparent suppression of astrocyte differentiation (Supplementary Fig. S6A, B). Next, we investigated whether *Ddx20*-deletion-mediated OPC loss is due to apoptosis or cell cycle arrest. We monitored cCasp3 expression and bromodeoxyuridine (BrdU) labeling, to investigate apoptosis and cell cycle arrest, respectively. The percentage of cCasp3-expressing Olig2-positive cells notably increased in *Ddx20* cKO spinal cords, whereas the ratio of BrdU-positive proliferating cells to Olig2-positive cells significantly decreased, compared with those in control spinal cords (Fig. 3I–L). Thus, these results suggested that OPCs lacking *Ddx20* in spinal cords lose their ability to maintain themselves, due to increased apoptosis and cell cycle arrest.

### *Ddx20* deficiency potentiates the p53 pathway

**Fig. 4:**
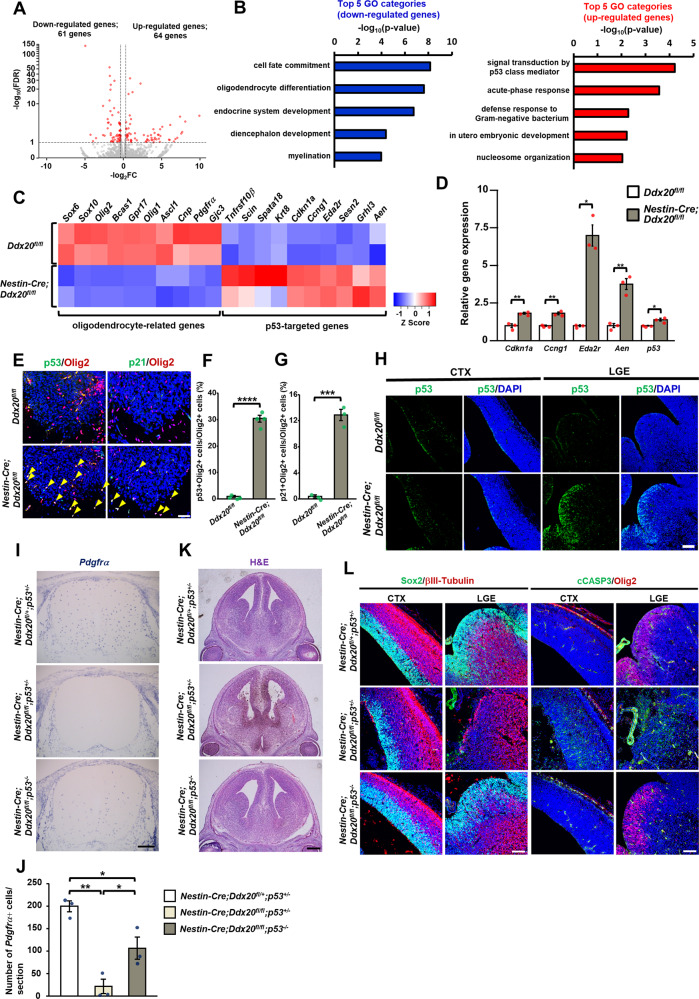
Activation of the p53 pathway in *Ddx20*-deficient CNS. **A** Volcano plot showing differentially expressed genes, in control versus *Nestin-Cre;Ddx20* cKO spinal cords, at E14.5. The fold change (*x*-axis) is plotted against significance [FDR, y-axis (semilogarithmic scale)]. The red-dots represent significantly low or high gene expression, compared with control levels. **B** Gene ontology (GO) analysis, using Metascape, shows the Top 5 terms among the GO terms for significantly decreased (blue bars) or increased (red bars) gene expression groups, compared with control expression levels. **C** Heat map, using normalized RPKM (reads per kilobase of exon per million mapped sequence reads) values (Z-scores) from RNA-seq data, depicts oligodendrocyte- and p53 pathway-related genes with significant differences in expression levels between control and *Nestin-Cre;Ddx20* cKO mice. **D** RT-qPCR for p53 target genes, to validate the RNA-seq data, in control and *Nestin-Cre;Ddx20* cKO spinal cords, at E14.5. *n* = 3 mice per group. Housekeeping gene *Gapdh* was used as an internal control. **E** Immunohistochemistry for p53, p21, and Olig2, in control and *Nestin-Cre;Ddx20* cKO mice, at E13.5. Yellow arrowheads indicate cells that are double-positive for Olig2 and each marker. The percentage of p53- or p21-positive cells among Olig2-positive cells, shown in **E**, respectively. *n* = 4 (**F**) or *n* = 3 (**G**) mice per group. **H** Immunohistochemistry for p53 in the telencephalons of control and *Nestin-Cre;Ddx20* cKO mice at E13.5. Images represent the mouse cerebral cortex (CTX, left images) and lateral ganglionic eminence (LGE, right images). **I** In situ hybridization for *Pdgfrα*, to investigate whether p53 ablation leads to the rescue of OPC loss in *Nestin-Cre;Ddx20* cKO spinal cords. **J** Bar chart showing the average number of *Pdgfrα*-positive cells, per section, in the spinal cords shown in **I**. Point indicates the average number of *Pdgfrα*-positive cells from three sections of each mouse spinal cord, at E14.5. *n* = 3 mice per group. **K** H&E staining to examine whether p53 ablation rescued the disruption of brain structures caused by *Ddx20* deficiency, in E17.5 mice. **L** Confocal images of the CTX and LGE of representative control, *Nestin;Cre;Ddx20* cKO, and *Nestin-Cre;Ddx20* cKO;*p53* KO brains, at E14.5. Immunohistochemistry for Sox2 and βIII-Tubulin (left images) or cCasp3 and Olig2 (right images). Bar charts represent –log_10_ (*p*-value) (**B**), the mean ± SD. (**D**, **F**, and **G**), and the mean ± SEM. (**J**). Statistical analysis was performed by two-tailed, unpaired *t*-test (**D**, **F**, and **G**) and one-way ANOVA, with post hoc Tukey’s test (**J**). *p < 0.05; **p < 0.01; ***p < 0.001; ****p < 0.0001. Scale bars, 50 μm (E); 100 µm (H); 200 μm (I); 400 μm (K); 100 μm (L).

To identify gene expression profiles associated with loss of NPCs and OPCs in *Ddx20*-deficient mice, we performed a transcriptome analysis of mouse embryonic spinal cords, using RNA sequencing (RNA-seq, see Supplementary Table S2). Total RNA was isolated from the E14.5 spinal cords of two littermate control (*Ddx20*^*flox/flox*^) and CNS-specific Ddx20 cKO mice (*Nestin-Cre;Ddx20*^*flox/flox*^). RNA-seq analyses identified 125 genes with significantly different expression levels between control and *Ddx20* cKO mice, including 61 downregulated genes (fold change <0.8) and 64 upregulated genes (fold change > 1.2) in *Ddx20* cKO mice compared with control mice, with a false discovery rate (FDR) < 0.1 and *p* < 0.001 (Fig. 4A and Supplementary Table S3). Gene ontology (GO) analysis showed that genes associated with the p53-mediated apoptosis pathway had the highest fold change among the upregulated gene set (Fig. 4B, C and Supplementary Tables S4, S5). Reverse transcriptase-quantitative polymerase chain reaction (RT-qPCR) confirmed the significant upregulation of p53 target genes in *Ddx20* deficient spinal cords (Fig. 4D). To investigate whether the p53 pathway was upregulated in oligodendrocyte lineage cells, immunohistochemistry was performed, using p53, p21, and Olig2 antibodies. *Ddx20* ablation in the CNS and OPCs drastically increased the ratios between p53- and p21-positive cells and Olig2-positive cells (Fig. 4E–G, and Supplementary Fig. S7A–C). Increased p53 levels were also detected in the embryonic cerebral cortex and ganglionic eminence of *Ddx20* cKO mice (Fig. 4H). To directly assess whether the activation of the p53 pathway is associated with NPC and OPC loss, *Ddx20* and *p53* double-knockout mice (*Nestin-Cre;Ddx20*^*flox/flox*^;*p53*^*−/−*^ mice) were generated. We collected E14.5 embryos and counted the number of *Pdgfrα*-expressing OPCs. OPC loss in the *Ddx20* cKO spinal cord was significantly rescued in a *p53*-null background (Fig. 4I, J). In addition, *p53*-null mice also showed the near-complete rescue of apoptosis in Sox2-positive NPCs in the brain, at E14.5 (Fig. 4K, L). These data indicated that p53 activation is likely the primary cause of NPC and OPC defects in *Ddx20* cKO mice, at this stage.

### DNA damage induction and the splicing dysregulation of *Mdm2* mRNA in *Ddx20*-deficient mice

**Fig. 5:**
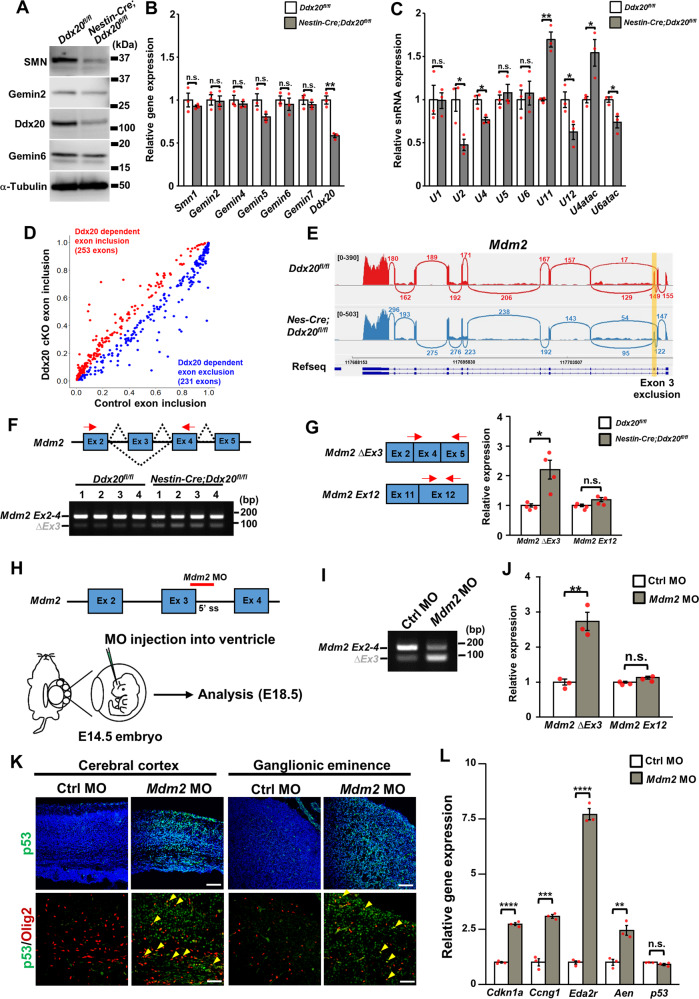
Splicing dysregulation of *Mdm2* mRNA in CNS-specific *Ddx20* cKO mice. **A** Western blotting for SMN, Gemin2, Ddx20, Gemin6, and α-Tubulin in Control and *Nestin-Cre; Ddx20* cKO spinal cords at E13.5. α-Tubulin was used as a loading control. **B** RT-qPCR for components of the SMN complex in control and *Nestin-Cre;Ddx20* cKO spinal cords at E14.5. *Actb* gene was used as an internal control. *n* = 3 mice per group. **C** RT-qPCR for spliceosomal U snRNAs in control and *Nestin-Cre;Ddx20* cKO spinal cords at E14.5. 5S rRNA was used as an internal control. *n* = 3 mice per group. **D** Scatter plot represents the rate of exon inclusion in control and *Nestin-Cre;Ddx20* cKO mRNA-seq significant hits. Each point indicates the mean, obtained from two biological replicates, for an individual alternative splicing event. **E** Sashimi plot showing the differential usage of *Mdm2* exon 3 between control and *Nestin-Cre;Ddx20* cKO mice. **F** Semi-quantitative RT-PCR for the alternative splicing of *Mdm2* exon 3, in control and *Nestin-Cre;Ddx20* cKO mice. *n* = 4 mice per group. Arrows indicate the *Mdm2* mRNA primer recognition sites. **G** RT-qPCR for the exclusion of *Mdm2* exon 3, in control and *Nestin-Cre;Ddx20* cKO mice. Arrows indicate the *Mdm2* mRNA primer recognition sites. *n* = 4 mice per group. *Actb* gene was used as an internal control. **H** Schematic of splice-modifying MOs, targeting the 5’ splice site of *Mdm2* exon 3, and the induction strategy for in vivo *Mdm2* splice-switching. MOs were injected into the ventricles of E14.5 mouse brains. Ninety-six hours after injection, the embryos were analyzed. **I** RT-PCR gel images showing the effective induction of *Mdm2* exon 3 splice-skipping, in control and *Mdm2* MO-injected brains, at E18.5. **J** RT-qPCR for the splice-skipping of *Mdm2* exon 3, in control and *Mdm2* MO-injected brains. *n* = 3 mice per group. *Actb* gene was used as an internal control. **K** Double-immunostaining for p53 and Olig2 in the cerebral cortex and ganglionic eminence of control and *Mdm2* MO-injected brains. Yellow arrowheads indicate cells double-positive for p53 and Olig2. **L** RT-qPCR for p53-related genes in control and *Mdm2* MO-injected brains. *n* = 3 mice per group. *Actb* gene was used as an internal control. Bar charts represent the mean ± SD. Statistical analysis was performed by two-tailed, unpaired *t*-test. *p < 0.05; **p < 0.01, ***p < 0.001, ****p < 0.0001; n.s., not significant.

To investigate the mechanism underlying p53 activation in *Ddx20* cKO mice, we performed immunohistochemistry for the phosphorylated H2A histone family member X (γH2AX), a marker of DNA damage. CNS-specific *Ddx20* ablation showed the drastic appearance of γH2AX-positive cells in brains and spinal cords (Supplementary Fig. S8A–C). In contrast, no significant differences were observed for the ratio of γH2AX-positive cells among Olig2-positive cells in OPC-specific *Ddx20* cKO spinal cords (Supplementary Fig. S8D, E), despite p53 activation (Supplementary Fig. S7A–C). These results indicated that both DNA damage-dependent and -independent mechanisms trigger p53 activation in *Ddx20* mutants. Therefore, we searched for DNA damage-independent mechanisms associated with p53 activation. We found a significant decrease in SMN protein levels but not its gene expression levels in spinal cords of *Ddx20* cKO mice (Fig. 5A, B). Given that Ddx20 directly binds to SMN [25], these results suggest that Ddx20 contributes to SMN stability. Furthermore, the expression of some spliceosomal U snRNAs was significantly altered in *Ddx20* cKO mice (Fig. 5C), suggesting that *Ddx20* ablation leads to defects of the SMN complex and dysregulation of snRNP assembly, which is an essential process for RNA splicing. Based on these results, to investigate the effects of *Ddx20* cKO on RNA splicing, the above-described RNA-seq data (Fig. 4) were analyzed at the exon junction level, revealing that 231 alternative exon exclusions and 253 alternative exon inclusions were significantly induced in *Ddx20*-deficient spinal cords compared with control spinal cords, with an FDR < 0.1 and *p* < 0.001 (Fig. 5D). These alternative exon changes (totaling 484 events) can be categorized into 6 types: cassette exons, mutually exclusive exons, tandem cassette exons, alternative 5′ site, alternative 3′ site, and intron retention (Supplementary Fig. S9A). Among these, 113 significant alternative splicing changes in 75 genes were detected between control and *Ddx20* cKO (|Δ*I* | > 0.1) mice (Supplementary Table S6). GO analysis demonstrated that splicing-related terms and cell cycle-related terms were highly ranked (Supplementary Fig. S9B and Supplementary Table S7). We identified the occurrence of exon 3 skipping in *Mdm2*, which encodes an E3 ubiquitin ligase associated with p53 protein degradation (Fig. 5E). The semi-quantitative RT-PCR and RT-qPCR validation of RNA-seq data confirmed the aberrant exon 3 exclusion from *Mdm2* mRNA, without any changes in the total *Mdm2* mRNA expression level between control and *Ddx20* mutants (Fig. 5F, G). Exon 3 of *Mdm2* encodes a p53-binding domain, and the deletion of this domain results in the accumulation and activation of p53 [26]. To investigate whether the deletion of the endogenous *Mdm2* exon 3 is sufficient to facilitate p53 activation in the CNS, we induced exon 3 skipping by injection morpholino antisense oligos (MO) [20] into the embryonic mouse brains. *Mdm2* MO was injected at E14.5, and we monitored exon 3 skipping of *Mdm2* mRNA and p53 activation at E18.5 (Fig. 5H–L). We first confirmed the effective exclusion of *Mdm2* exon 3, using RT-PCR (Fig. 5I, J). Immunostaining for p53 showed that *Mdm2* MOs drastically and extensively facilitated increased p53 levels in the cerebral cortex and ganglionic eminence (Fig. 5K). The accumulation of p53 was also identified in Olig2-positive cells (Fig. 5K). The significant upregulation of p53 target genes was also confirmed by RT-qPCR, in *Mdm2* MO-injected brains (Fig. 5L). These data indicated that the splicing dysregulation of *Mdm2* exon 3 leads to p53 activation.

### Olig2 regulates the p53 activation through the stabilization of Ddx20 proteins

**Fig. 6:**
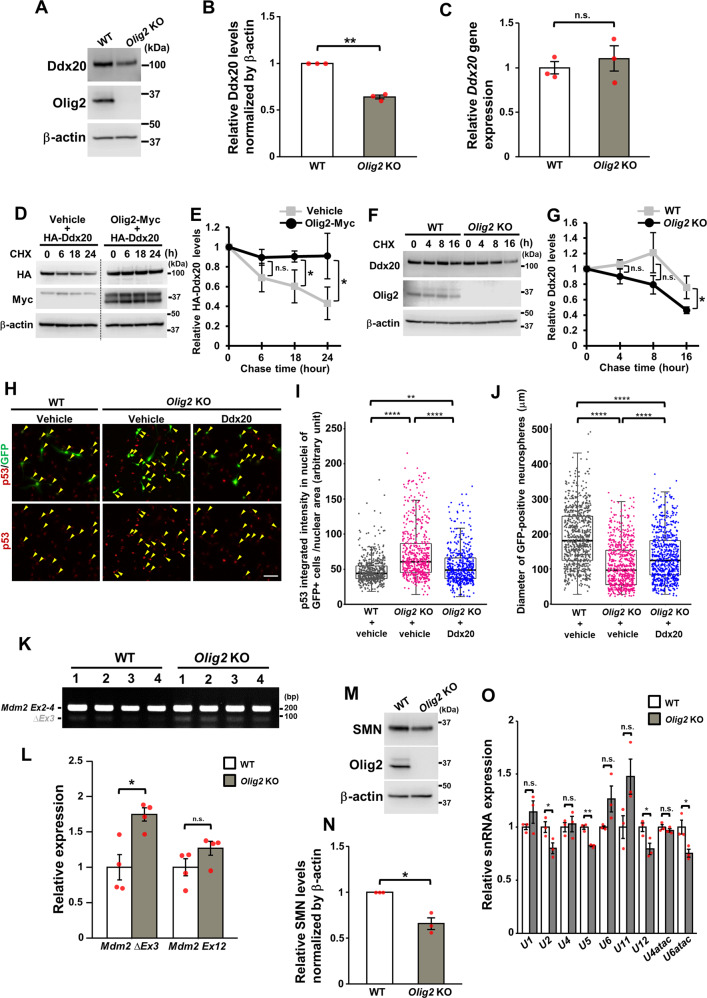
Olig2 stabilizes Ddx20 proteins and promotes NSC proliferation, through the suppression of p53 accumulation. **A, B** Western blotting for Ddx20 and Olig2, in WT and *Olig2*-KO neurospheres at E14.5. β-actin was used as a loading control. The ratio of Ddx20 protein levels in *Olig2*-KO neurospheres to those in WT neurospheres. The density of Ddx20 was normalized against that for β-actin. *n* = 3 biologically independent experiments. **C** RT-qPCR for Ddx20 in WT and *Olig2*-KO neurospheres. *n* = 3 mice per group. *Actb* gene was used as an internal control. **D** Cycloheximide (CHX) chase assay, showing Ddx20 degradation in Plat-E cells coexpressing HA-Ddx20 with or without Olig2-Myc. The cells were treated with CHX (200 μg/ml) for 6, 18, and 24 h, (0 indicates no treatment), and then western blotting against HA, Myc, and β-actin was performed. β-actin was used as loading controls. **E** A line graph showing the densitometric data of the HA-Ddx20 signal from the western blotting experiment in **D**. The vertical axis represents the ratio of the HA-Ddx20 signals in each CHX treatment condition to the HA-Ddx20 signal at the start of the chase. The density of HA-Ddx20 was normalized against that for β-actin. *n* = 3 biologically independent experiments. **F** CHX chase assay, showing Ddx20 degradation in WT and *Olig2*-KO NPCs. The cells were treated with CHX for 4, 8, and 16 h, (0 indicates no treatment), and then western blotting against Ddx20, Olig2, and β-actin was performed. The density of Ddx20 was normalized against that for β-actin. **G** A line graph showing the densitometric data of the Ddx20 signal from the western blotting experiment in **D**. The vertical axis represents the ratio of the Ddx20 signals in each CHX treatment condition to the Ddx20 signal at the start of the chase. *n* = 3 biologically independent experiments. **H** Double-immunostaining for p53 and GFP, in WT and *Olig2-*KO NPCs, infected with control or *Ddx20*-retrovirus vectors, respectively. Yellow arrowheads indicate cells that are double-positive for p53 and GFP. Scale bar, 50 μm. **I** Box plots (center, median; box, interquartile range; whiskers, 1.5x interquartile range) and dot plots represent the intensity of p53 in the nucleus of each GFP-positive cell. Data were pooled from three biologically independent experiments. At least 150 GFP-positive cells, in each condition, from each experiment, were analyzed. WT + vehicle (gray), *n* = 540; *Olig2*-KO + vehicle (pink), *n* = 592; *Olig2*-KO + Ddx20 (blue), *n* = 500. **J** Box plots and dot plots represent the diameter of each GFP-positive neurosphere. Data were pooled from three biologically independent experiments. At least 120 GFP-positive neurospheres, in each condition, from each experiment, were analyzed. WT + vehicle (gray), *n* = 734; *Olig2*-KO + vehicle (pink), *n* = 728; *Olig2*-KO + Ddx20 (blue), *n* = 850. **K** Semi-quantitative RT-PCR for the detection of *Mdm2* exon 3 alternative splicing in neurospheres derived from WT or *Olig2-*KO brains. *n* = 4 mice per group. **L** RT-qPCR analysis for the detection of *Mdm2* exon 3 skipping and total *Mdm2* expression, in WT and *Olig2-*KO neurospheres. *n* = 4 mice per group. *Actb* gene was used as an internal control. **M**, **N** Western blotting for SMN, Olig2 and β-Actin in WT and *Olig2*-KO neurospheres at E14.5. The ratio of Ddx20 protein levels in *Olig2*-KO neurospheres to those in WT neurospheres. The density of Ddx20 was normalized against that for β-Actin. *n* = 3 biologically independent experiments. **O** RT-qPCR for spliceosomal U snRNAs in WT and *Olig2-*KO neurospheres at E14.5. 5 S rRNA was used as an internal control. *n* = 3 mice per group. Bar charts and line plots represent mean ± SD. Statistical analysis was performed by two-tailed, unpaired *t* test (**B**, **C**, **E**, **J**, **K**, and **L**) and Kruskal-Wallis test, with a post hoc Steel-Dwass test (**G** and **H**). *p < 0.05; **p < 0.01; ****p < 0.0001; n.s., not significant.

**Fig. 7:**
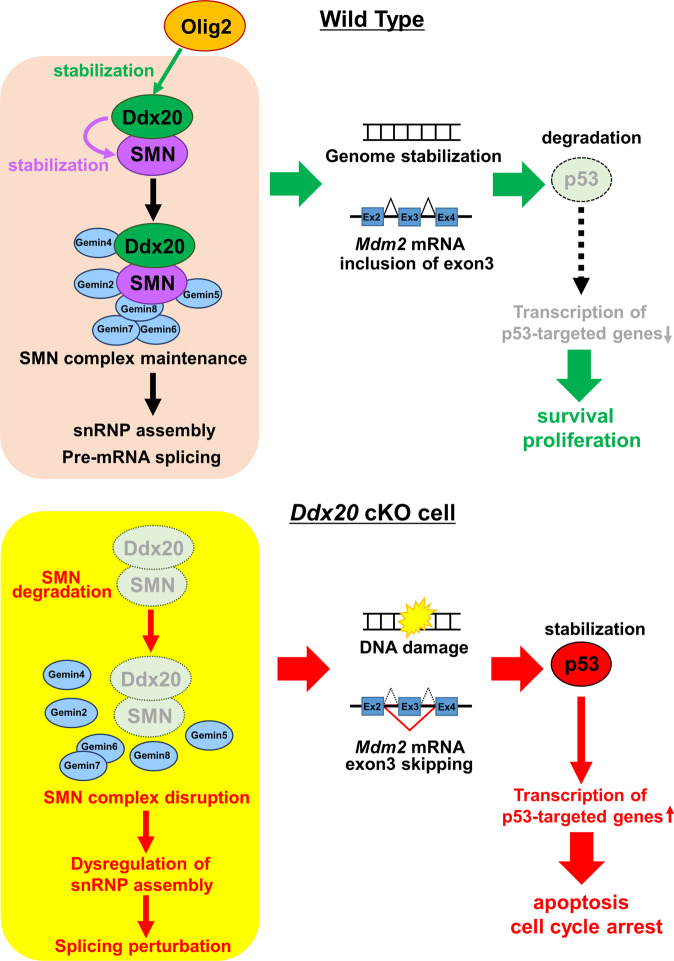
Olig2-Ddx20 axis-mediated suppression of p53 pathway. Schematic drawing the model of this study. *Ddx20* deletion leads to SMN degradation and dysregulation of snRNA repertoires, indicating that Ddx20 contributes to SMN complex maintenance and snRNP assembly. Ddx20 ablation also promotes the p53 stabilization and the expression of its target genes through DNA damage and splicing dysregulation of *Mdm2* mRNA, resulting in apoptosis and cell cycle arrest in NPCs and OPCs. These results suggest that Ddx20 suppresses the p53 pathway through genome stabilization and splicing regulation of *Mdm2* mRNA. Importantly, Olig2-mediated Ddx20 stabilization contributes to SMN complex maintenance and normal snRNP assembly, leading to the inhibition of p53-mediated apoptosis and cell cycle arrest in NPCs.

Next, we investigated the effects of Olig2 on the Ddx20 function. To examine whether Olig2 is involved in the stabilization of Ddx20 in NPCs, the levels of Ddx20 protein and *Ddx20* mRNA were assessed by western blotting and RT-qPCR, respectively, in neurospheres, derived from wild-type (WT) and *Olig2-*null mice. Ddx20 levels were significantly reduced in *Olig2*-null neurospheres compared with those in WT neurospheres (Fig. 6A, B), whereas *Ddx20* mRNA levels were not significantly different between the two genotypes (Fig. 6C). To further confirm Olig2-mediated Ddx20 stabilization, we performed the protein stability assay with cycloheximide, a protein translation inhibitor, in Plat-E cells coexpressing exogenous Ddx20 with or without Olig2. The results showed that Olig2 significantly enhanced the Ddx20 stability (Fig. 6D, E). In addition, the enhanced degradation of Ddx20 proteins was observed in *Olig2*-deficient NPCs compared with WT NPCs, in the presence of cycloheximide (Fig. 6F, G), indicating that Olig2 contributes to Ddx20 protein stabilization. Olig2 ablation has been reported to induce p53 stabilization, reducing NPC proliferation [27, 28]. Therefore, we examined whether the progression of Ddx20 degradation in *Olig2*-deficient NPCs affects p53 stabilization. We exogenously expressed Ddx20 in *Olig2*-deficient NPCs and examined p53 expression using immunohistochemistry. Cultured WT and *Olig2*-deficient NPCs were transduced with Ddx20 retroviruses, including IRES-GFP, and then immunocytochemistry was performed to examine p53 and GFP expression levels. The forced expression of *Ddx20* in *Olig2*-deficient NPCs significantly suppressed the increase in the percentage of cells expressing high levels of p53 among GFP-expressing cells (Fig. 6H, I). Furthermore, *Ddx20* overexpression partially rescued the Olig2-loss-mediated attenuation of neurosphere size, suggesting that Ddx20 ameliorated the proliferative capacity of NPCs (Fig. 6J). Finally, we confirmed the exclusion of *Mdm2* exon 3 in *Olig2*-deficient NPCs, whereas no significant difference in the total *Mdm2* mRNA expression levels was observed between WT and *Olig2*-null NPCs (Fig. 6K, L). Forced expression of exon 3-containing Mdm2 partially suppressed p53 stabilization in *Olig2*-deficient NPCs, indicating that splicing dysregulation of *Mdm2* is involved in p53 stabilization in *Olig2*-deficient NPCs (Supplementary Fig. S10A, B). Furthermore, consistent with *Ddx20* cKO mice, *Olig2* KO demonstrated a significant decrease of SMN protein levels and dysregulation of some U snRNAs (Fig. 6M–O). Taken together, these results suggested that Olig2 stabilizes Ddx20 proteins, suppressing p53 activation, at least partially, through the maintenance of SMN complex and the regulation of *Mdm2* splicing (Fig. 7).

## Discussion

This study demonstrated that *Ddx20* deficiency resulted in p53 activation through dysregulation of *Mdm2* splicing and DNA damage (Fig. 7). Ddx20 is involved in spliceosome formation, as a component of the SMN complex [10]. Interestingly, spinal muscular atrophy (SMA) model mice (*Smn1*^*−/−*^*; SMN2* transgenic mice) demonstrated p53 activation [29] through the dysregulation of *Mdm2* and *Mdm4* splicing in motor neurons [20]. Considering that *Ddx20* ablation leads to the severe degradation of SMN (Fig. 5A, B) and defects in snRNP assembly (Fig. 5C) [30], Ddx20 likely plays a key role in the splicing regulation of *Mdm2*. Furthermore, *Ddx20* deletion facilitated DNA damage in NPCs and OPCs. One possible mechanism is the formation of inappropriate DNA-RNA hybrids, known as R-loops, during the transcription process, which increases genome instability and results in DNA damage [31]. The generation of R-loops is normally prevented by various ‘guardians’, such as topoisomerase, RNase H, and mRNP biogenesis. The SMN complex, containing Ddx20, contributes to the assembly of some mRNPs [32], and *SMN* disruption has been reported to result in the generation of R-loops [29]. Therefore, Ddx20 may also be involved in the removal of R-loops.

In our study, *Ddx20* deficiency-induced SMN degradation and defect of snRNP assembly. Given that Ddx20 directly binds to SMN and SMN downregulation also leads to a decrease in Ddx20 levels [25, 30], it is plausible that Ddx20 and SMN are interdependent for protein stabilization and snRNP assembly. Our in vivo findings provide conclusive evidence that Ddx20 is a strong stabilizer for SMN and highlight the significant role of Ddx20 in the SMN complex. Importantly, SMN proteins with mutations found in SMA patients show a significantly reduced interaction with Ddx20 [25, 33], suggesting that the Ddx20-SMN interaction and the stabilization of both proteins are critical for proper function of SMN complexes.

As discussed above, *Ddx20* mutant mice (this study) and *Smn1* mutant mice showed commonality, in terms of DNA damage and p53 pathway activation; however, phenotypic differences were also observed. *Ddx20*-null mice and *Smn1-*null mice die at different stages, with *Ddx20*-null embryos demonstrating lethality as early as the four-cell stage [14], whereas *Smn1*-null embryos show lethality after the morula stage [34]. Furthermore, SMA model mice (*Smn1*^*−/−*^*; SMN2* mice) do not demonstrate changes in OPC proliferation and/or oligodendrocyte differentiation [35]. These differences suggest that Ddx20 plays other roles, in addition to acting as a component of the SMN complex. Ddx20 has been suggested to have pleiotropic functions because it binds not only to components of the SMN complex but also to many factors including transcription factors [11, 36–38]. Thus, Ddx20 is involved not only in RNA splicing but also in transcriptional regulation, miRNA generation, translational regulation, and signaling regulation.

Our findings propose that the Olig2-Ddx20-p53 axis contributes to the maintenance of NPC proliferation. Olig2 has been reported to promote NPC proliferation, through the direct suppression of *p21* promoter-mediated transcription, which is a p53 target gene. In addition, Olig2 is phosphorylated at S10, S13, and S14, in NPCs, and counteracts p53 activation through the inhibition of p53 acetylation [27]. Altogether, our study provided evidence that Olig2 suppresses the p53 pathway through multiple mechanisms. In NPCs, the Olig2-Ddx20 interaction plays a role in proliferation and survival, whereas, in OPCs, Olig2 has been reported to be involved in the promotion of migration and differentiation, instead of proliferation [39, 40]. Therefore, the Ddx20-p53 axis in OPCs may function independently of Olig2. Olig2 has been reported to change binding partners during development, depending on its phosphorylation state, which contributes to neuron-oligodendrocyte fate switching in the pMN domain [41]. Similarly, the interaction between Olig2 and Ddx20 may be affected by the different molecular environments, including differences in Olig2 post-translational modifications, between NPCs and OPCs.

Although both Ddx20 and Olig2 deficiency affect the snRNA expression via SMN degradation, the type of variable snRNAs and the patterns of variation in snRNA expression are different between *Ddx20* cKO and *Olig2* KO mice. Considering that Zhang et al. demonstrated that the SMA mice showed a different repertoire of snRNAs that fluctuate depending on tissue and age [42], the difference between the two results may be due to the different tissue and cell populations. In this study, total RNAs were extracted from spinal cords of *Ddx20* cKO mice or neural progenitor cells cultured as neurospheres from *Olig2* KO mice, respectively. Various factors such as transcription, processing, transport, assembly, modification, and turnover rate of snRNAs may be different in *Ddx20* cKO spinal cord and *Olig2* KO neural progenitor cells. In addition, it is reported that the degree of SMN fluctuation correlates with the degree of snRNP assembly perturbation [43]. In *Olig2* KO mice, SMN expression is affected via destabilization of Ddx20, which may be a more indirect effect than in *Ddx20* cKO mice. Therefore, the effect of *Olig2* KO on the expression of snRNAs may be smaller than that of *Ddx20* cKO. These considerations may also explain the difference in snRNA expression between *Ddx20* cKO mice and the SMA model mice [42, 44], because the cellular composition of the *Ddx20* cKO spinal cord is different from that of the SMA spinal cord due to the rapid loss of OPCs and impairment of astrocyte differentiation.

Collectively, our studies demonstrate that Ddx20 is a crucial component for SMN complex formation and a potent suppressor of the p53 pathway, contributing to the maintenance of NSCs and OPCs during CNS development. Notably, a transcription factor Olig2 interacts with Ddx20, responsible for RNA splicing and miRNA synthesis, suggesting a broad influence of transcription factors on RNA metabolism. Furthermore, because Ddx20 has been implicated in cancer initiation and progression [38, 45], Ddx20 also may play an important role in the molecular basis underlying the promotion of glioma stem cell proliferation via Olig2-mediated repression of the p53 pathway [5, 28, 46, 47].

## Supplementary information

Supplementary Information

Supplementary Tables

## Data Availability

The data that support the findings of this study are available from the corresponding author upon request. RNA-seq raw data files were deposited at the DDBJ Sequenced Read Archive under the accession number DRA010555. The processed data of RNA-seq were deposited at the DDBJ Genomic Expression Archive (GEA) under the accession number E-GEAD-379.
