# Supplementary material for: Ddx20, an Olig2 binding factor, governs the survival of neural and oligodendrocyte progenitor cells via proper Mdm2 splicing and p53 suppression
Source: Cell Death Differ. 2022 Jan 1;29(5):1028–41. doi: 10.1038/s41418-021-00915-8 (PMC9090832; doi:10.1038/s41418-021-00915-8)
Supplement: Supplementary file 1 — Supplementary Information [file 41418_2021_915_MOESM1_ESM.docx]

**Supplementary information**

**Ddx20, an Olig2 binding factor, governs the survival of neural and oligodendrocyte progenitor cells via proper Mdm2 splicing and p53 suppression**

Norihisa Bizen^1^, Asim K. Bepari^1,2^, Li Zhou^1,3,4^, Manabu Abe^3,5^, Kenji Sakimura^3,5^, Katsuhiko Ono^6^, Hirohide Takebayashi^1,4*^

**Contents:**

**Supplementary Figures and Legends**

**Supplementary Materials and Methods**

**Supplementary References**

**Supplementary Tables**

**Supplemental figures**

Figure S1: Generation of *Ddx20* floxed mice and CNS-specific *Ddx20* KO mice.

Figure S2: Sox2-positive NPCs and motor neuron production are not severely affected in the CNS-specific *Ddx20* cKO spinal cords.

Figure S3: CNS-specific *Ddx20* cKO mice show slightly suppressed astrogliogenesis.

Figure S4: *Ddx20* deletion in Olig2-expressing cells results in the suppression of oligodendrocyte differentiation.

Figure S5: Generation of OPC-specific *Ddx20* cKO mice.

Figure S6; *Ddx20* deficiency in OPCs does not affect astogliogenesis.

Figure S7: The activation of p53 pathway in OPC-specific *Ddx20* deficient spinal cords.

Figure S8: CNS-specific ablation of *Ddx20* induces DNA damage in NPCs and OPCs.

Figure S9: Multiple types of alternative splicing are altered in CNS-specific *Ddx20* cKO spinal cords.

Figure S10: Exogenous expression of exon 3-containing Mdm2 alleviates p53 accumulation in *Olig2* deficient NPCs.

**
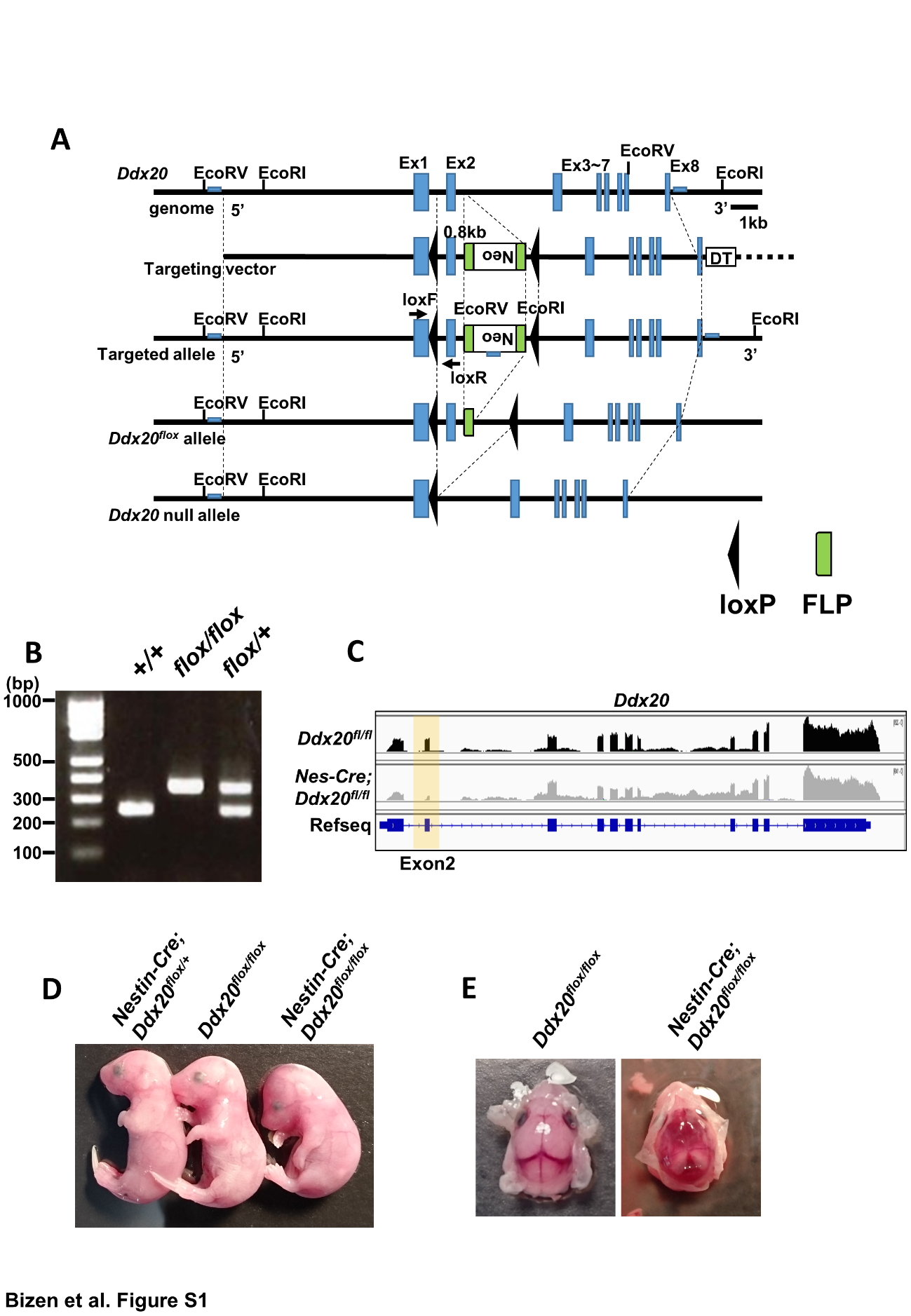
**

**Figure S1. Generation of *Ddx20* floxed mice and CNS-specific *Ddx20* KO mice.** (**A**) Schematic illustrating the strategy for the generation of *Ddx20* floxed mice and *Ddx20* KO mice. Construction of alleles and vectors for the mouse *Ddx20* genes: the wild type genome, targeting vector, flox-neo allele, floxed allele, and deleted allele. Arrows indicate primers for the distinction between the WT allele and the floxed allele. (**B**) Genotyping analysis for *Ddx20*-flox mice using RT-PCR: WT (*Ddx20^+/+^*), heterozygous (*Ddx20^flox/+^*), homozygous (*Ddx20^flox/flox^*) mice. (**C**) IGV (Integrative Genomics Viewer) image showing the mRNA expression of *Ddx20* from RNA-seq data in *Ddx20^flox/flox^* and *Nestin-Cre;Ddx20^flox/flox^* spinal cords at E14.5. The effective removal of Ddx20 exon2 in *Nestin-Cre;Ddx20^flox/flox^* mice is shown. (**D and E**) Gross morphology of Nestin-Cre;Ddx20^flox/flox^, Nestin-Cre;Ddx20^flox/+^, and Ddx20^flox/flox^ littermate at E18.5. Nestin-Cre;Ddx20^flox/flox^ mice showed a curled posture (D) and bleeding at the head region (E).

**
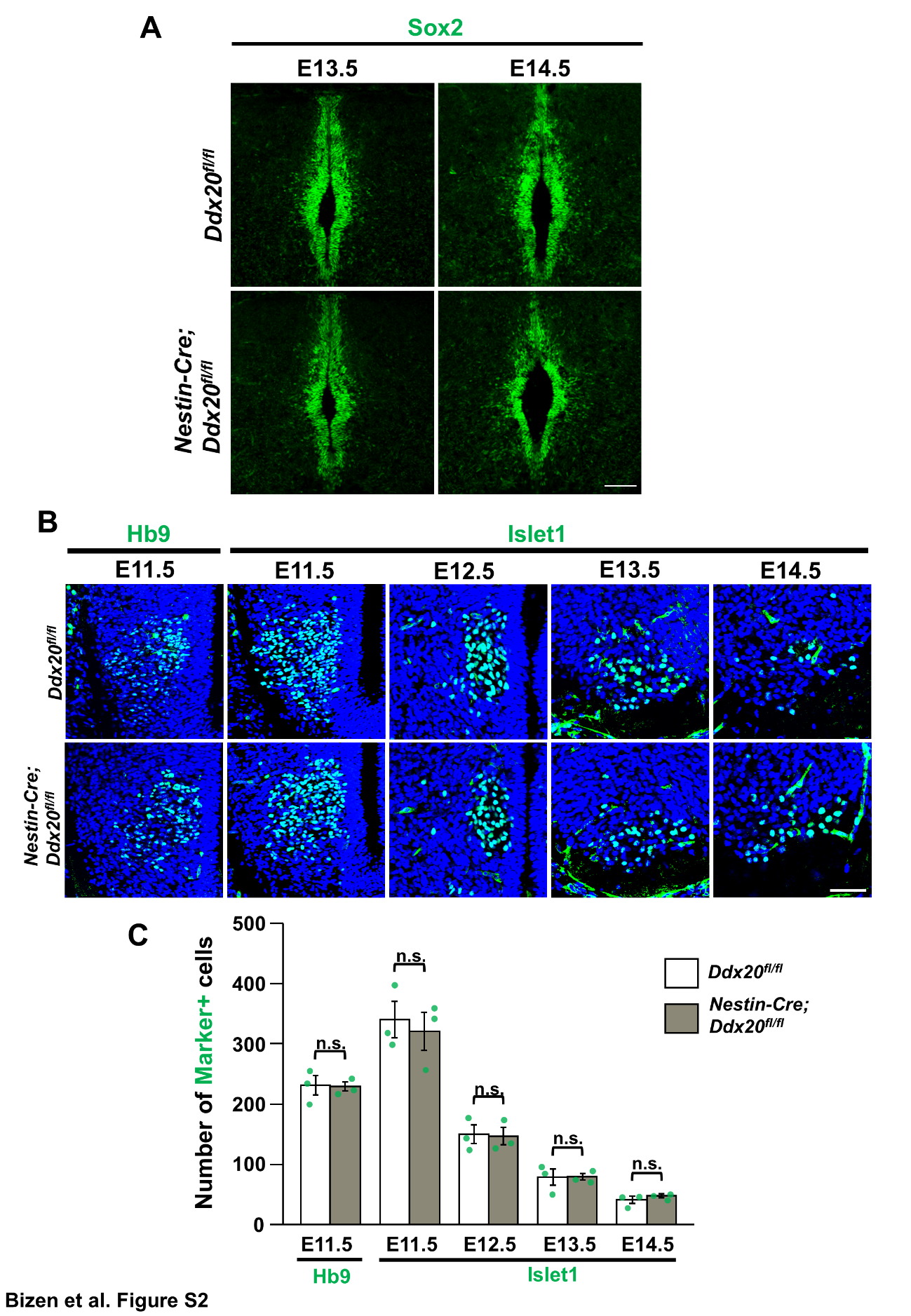
**

**Figure S2. Sox2-positive NPCs and motor neuron production are not severely affected in the CNS-specific *Ddx20* cKO spinal cords.** (**A**) Immunohistochemistry for Sox2 in spinal cords of control and *Nestin-Cre;Ddx20* cKO mice at E13.5 and E14.5. (**B**) Immunohistochemistry for Hb9 and Islet1 was performed to investigate the effect of *Ddx20* deficiency in the generation of motor neurons in the spinal cords of control and *Nestin-Cre;Ddx20* cKO mice at E11.5–14.5. No significant effect was observed at these stages. (**C**) A bar chart showing the average number of Hb9 or Islet1-positive cells per section in the spinal cords (three mice each for control and *Nestin-Cre;*Ddx20 cKO). Point indicates the average number of marker-positive cells in three sections of each mouse spinal cords at E11.5-E14.5. *n* = 3 mice per group. Bar plots represent mean ± SD. Statistical analysis was performed by two-tailed unpaired *t*-test. n.s., not significant. Scale bar, 100 μm (A); 50 μm (B).

**
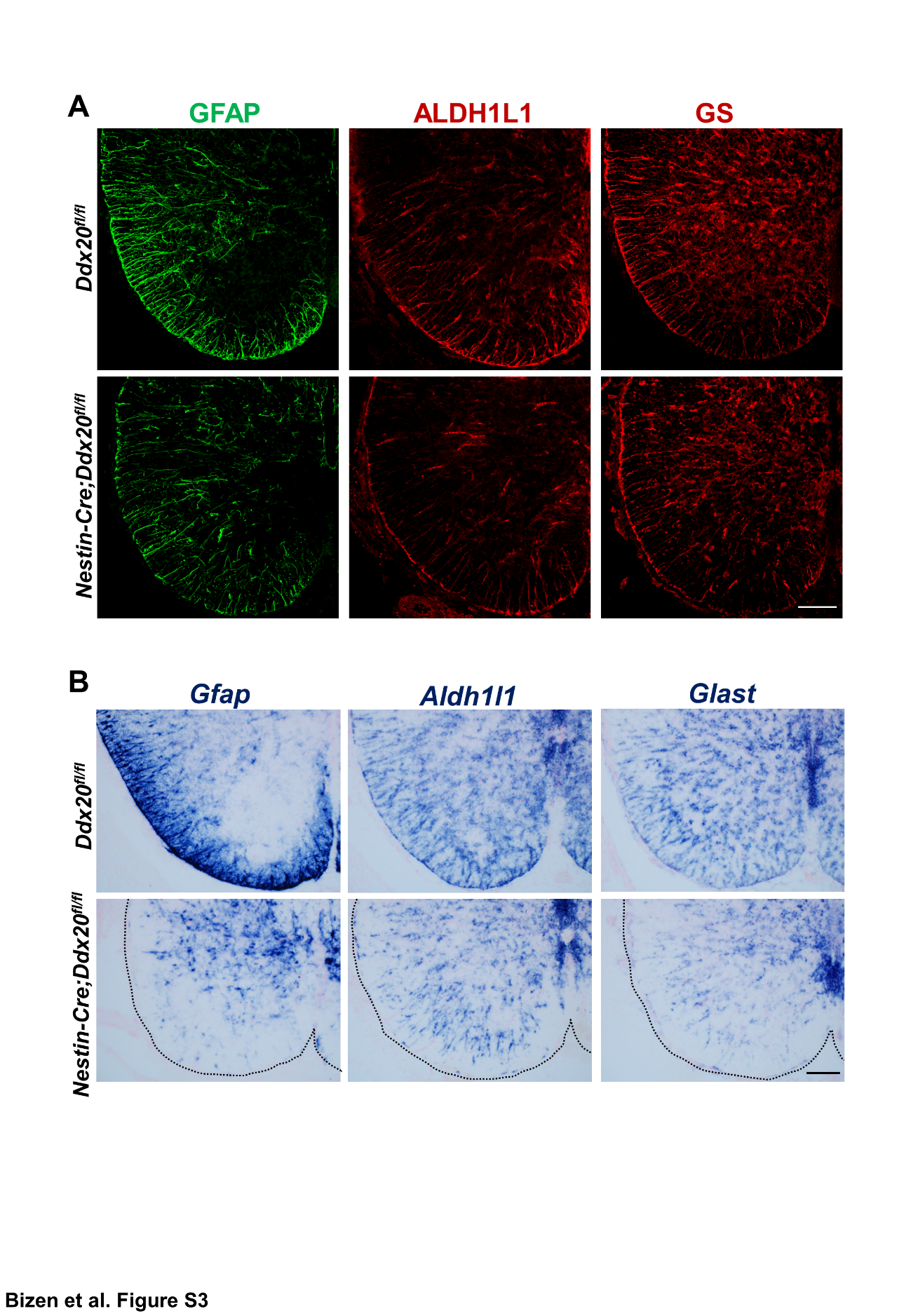
**

**Figure S3. CNS-specific *Ddx20* cKO mice show slightly suppressed astrogliogenesis.**

(**A, B**) Immunohistochemistry for GFAP, Aldh1l1, or GS (A) and in situ hybridization for *Gfap*, *Aldh1l1*, or *Glast* (B) to examine the influence of *Ddx20* ablation on the astrogliogenesis in the spinal cords of control and *Nestin-Cre;Ddx20* cKO mice at E17.5. Scale bars, 100 μm.

**
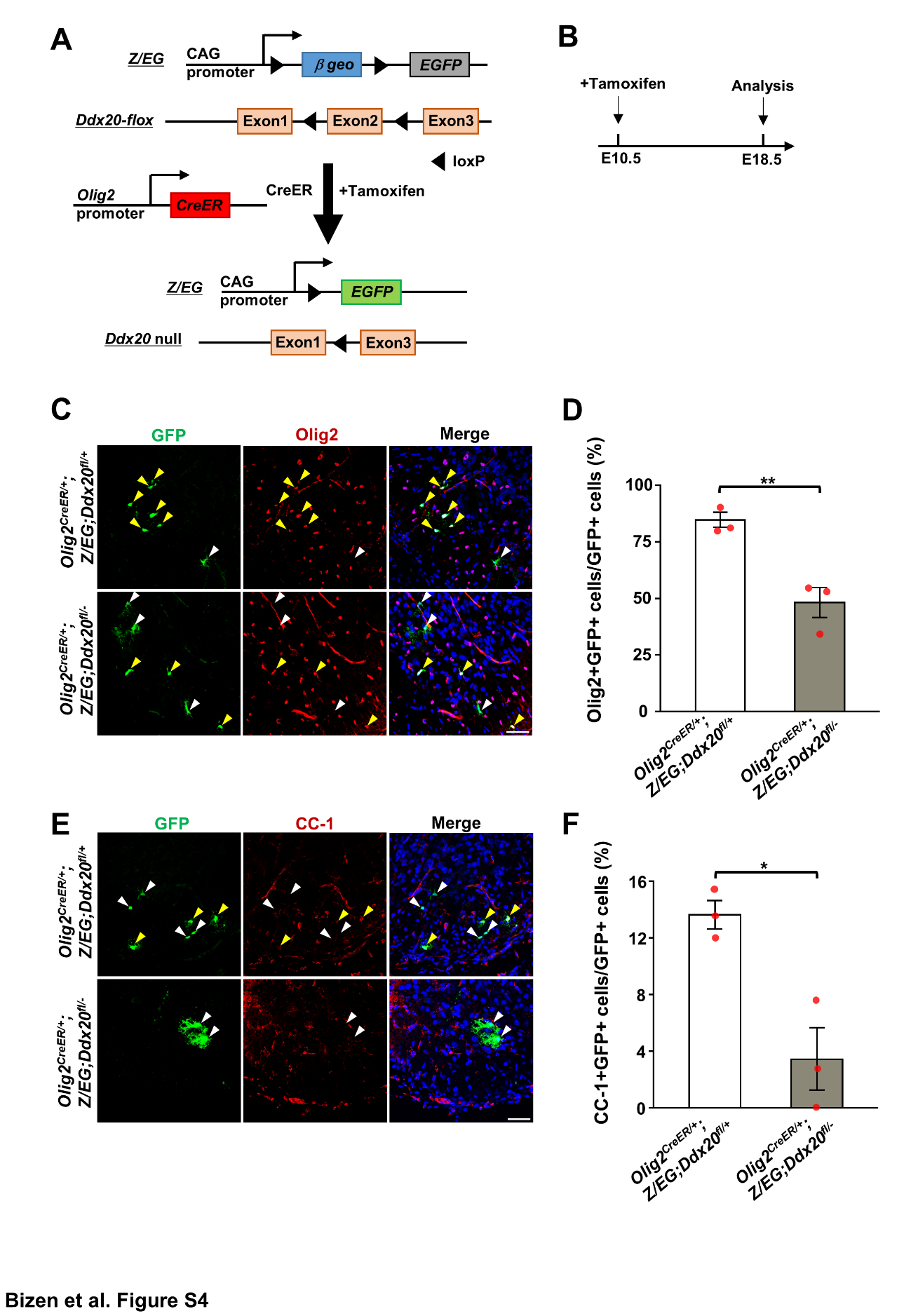
**

**Figure S4. *Ddx20* deletion in Olig2-expressing cells results in the suppression of oligodendrocyte differentiation.** (**A**) Schematic illustrating lineage tracing experiment of *Ddx20* deficiency in Olig2-expressing cells in the embryonic spinal cords. *Olig2^CreER/+^; Ddx20* cKO mice were analyzed to investigate the effect of *Ddx20* ablation in GFP- (Olig2-) expressing cells. (**B**) Tamoxifen was intraperitoneally injected into pregnant mice at E10.5, and then the embryos were analyzed at E18.5. (**C**–**F**) Double-immunostaining for GFP and Olig2 (C) or GFP and CC-1 (E) was performed in *Olig2^CreER/+^; Z/EG; Ddx20^flox/+^* and *Olig2^CreER/+^; Z/EG; Ddx20^flox/-^* spinal cords at E18.5. Yellow arrows indicate cells double-positive for Olig2 and GFP. The bar chart indicates the ratio of cells (in percentage) double-positive for GFP and each marker to the GFP-positive cells. *n* = 3 mice per group. Bar charts represent mean ± SD. Statistical analysis was performed by two-tailed unpaired *t*-test. *, *p* < 0.05; **, *p* < 0.01. Scale bars, 50 μm.

**
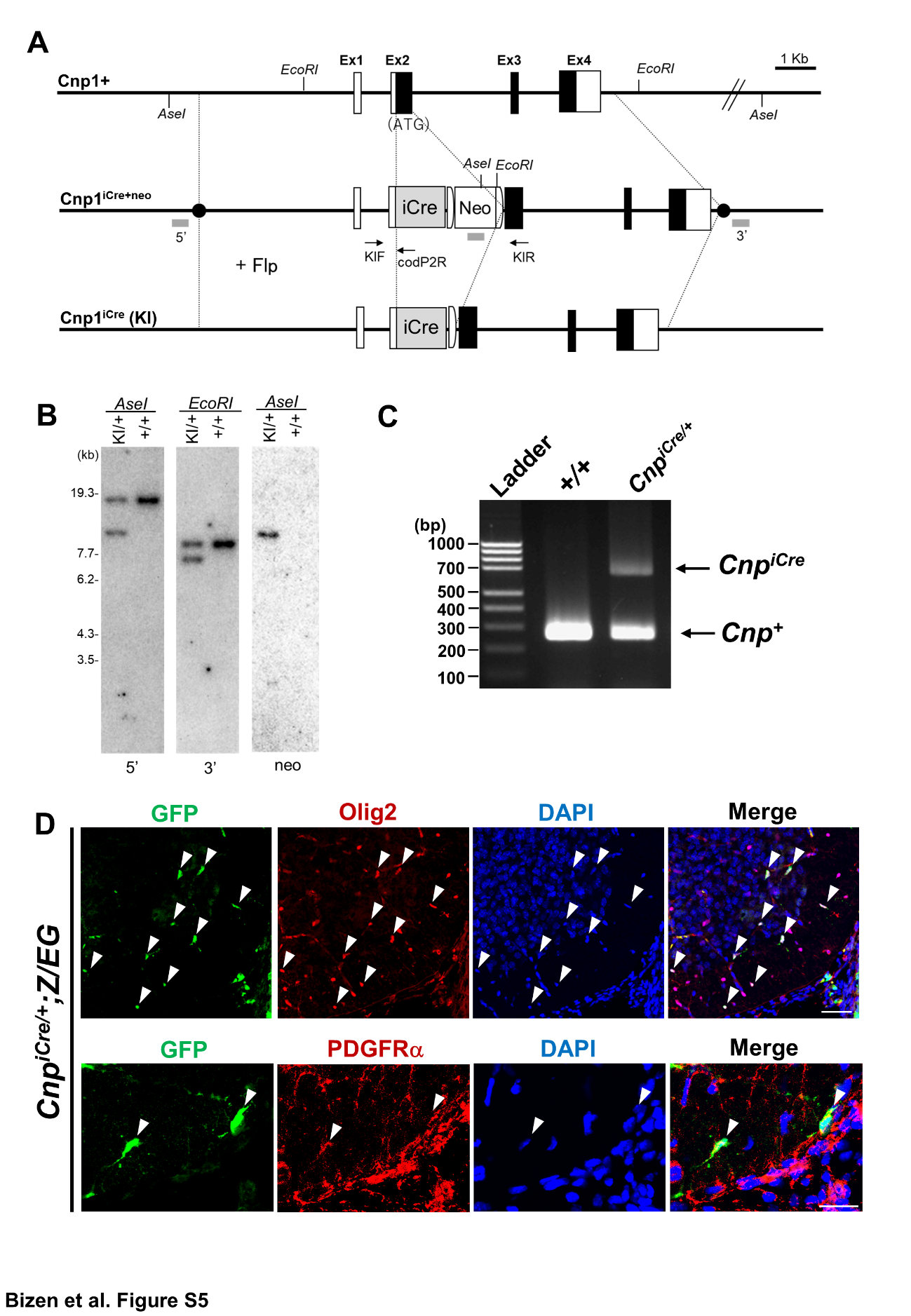
**

**Figure S5. Generation of OPC-specific *Ddx20* cKO mice.** (**A**) Schematic diagram of the gene targeting strategy. Top, original wild type allele. Middle, targeted construct. Bottom, expected genomic structure after Flp-mediated recombination. The 5' and 3' ends of homology arm (10.5 kb) of targeting vector are indicated as black circles. Position of 5', 3' and Neo DNA probes (gray line) and PCR primers (arrow) are shown. ATG is the translation initiation site of *Cnp1*. iCre, codon-improved Cre recombinase (*iCre*)-splice gene linked to WPRE-pA sequence (Gray box). Neo, PGK promotor-gb2-neomycin-resistant cassette flanked by two FRT elements (semicircles). (**B**) Confirmation of homologous recombination in ES cells. The image is a collage made from three Southern blotting filters. The molecular weight marker was used to adjust the position of bands. Bands are 18.0 kb (wt) and 10.4 kb (KI) with 5' probe; 9.0 kb (wt) and 7.7 kb (KI) with 3' probe; 10.4 kb (KI) with neo probe. Genomic DNA from original ES cells (+/+) and recombinant ES cells (KI/+) was digested with respective enzymes and hybridized with three different probes. (**C**) Genotyping PCR of WT and KI/+. The DNA size of bands are: WT, 250bp, KI, 658bp. The primers used for PCR genotyping are shown in (A). For primer sequences, see Supplementary Table 1. (**D**) Immunohistochemistry for GFP and Olig2 or GFP and PDGFRα positive cells in E14.5 spinal cords was performed to verify the recombination of *Cnp* promoter induced Cre recombination in OPCs. *Cnp^iCre/+^* mice were crossed with *Z/EG* reporter mice. White arrowheads indicate double-positive cells for GFP and Olig2, or GFP and PDGFRα. Scale bars, 50 μm (upper images); 25 μm (bottom images).

**
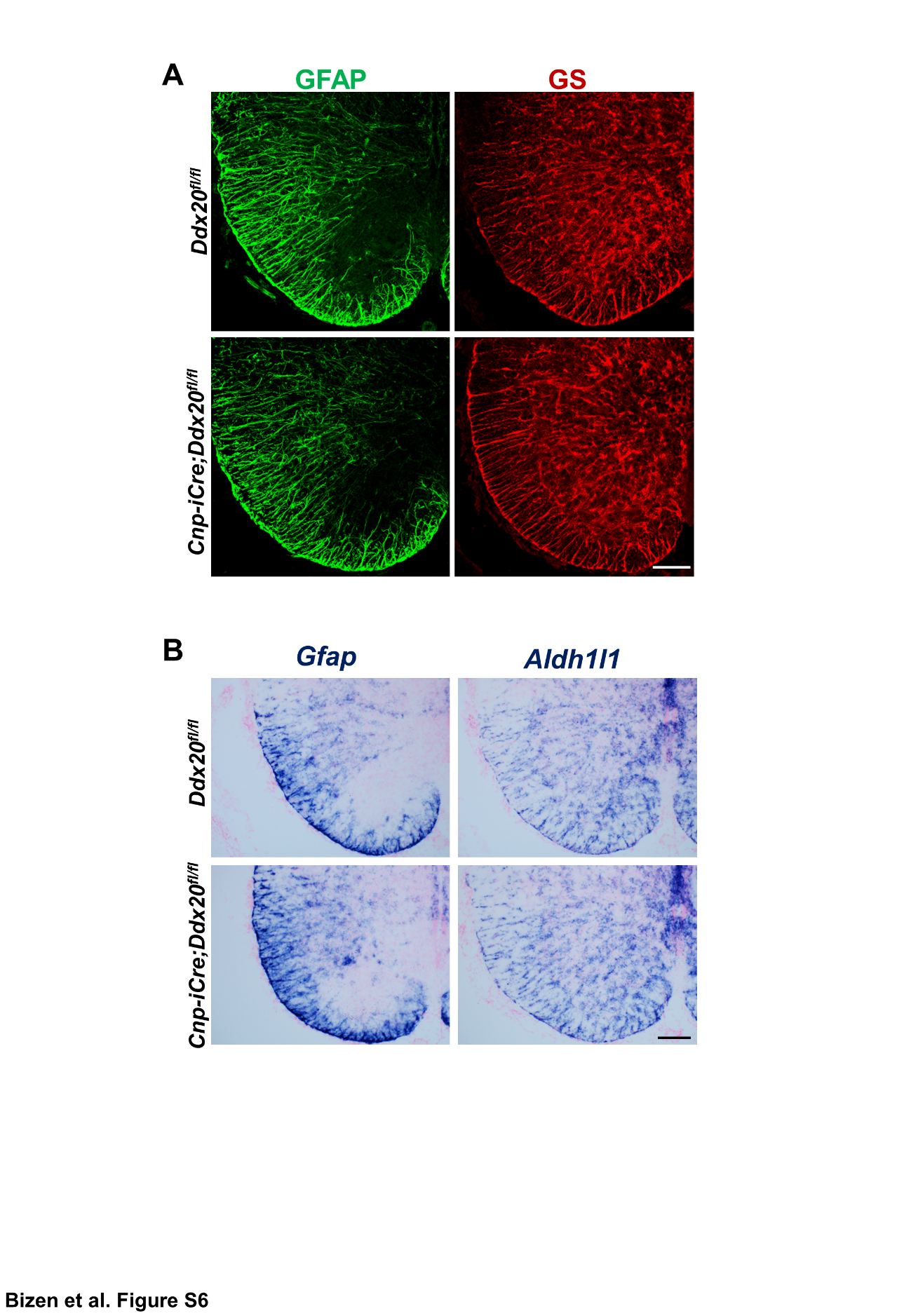
**

**Figure S6. *Ddx20* deficiency in OPCs does not affect astogliogenesis.**

(**A, B**) Immunohistochemistry for GFAP or GS (A) and in situ hybridization for *Gfap* or *Aldh1l1* (B) to examine the influence of *Ddx20* ablation on the astrogliogenesis in the spinal cords of control and *Cnp-iCre;Ddx20* cKO mice at E17.5. Scale bars, 100 μm.

**
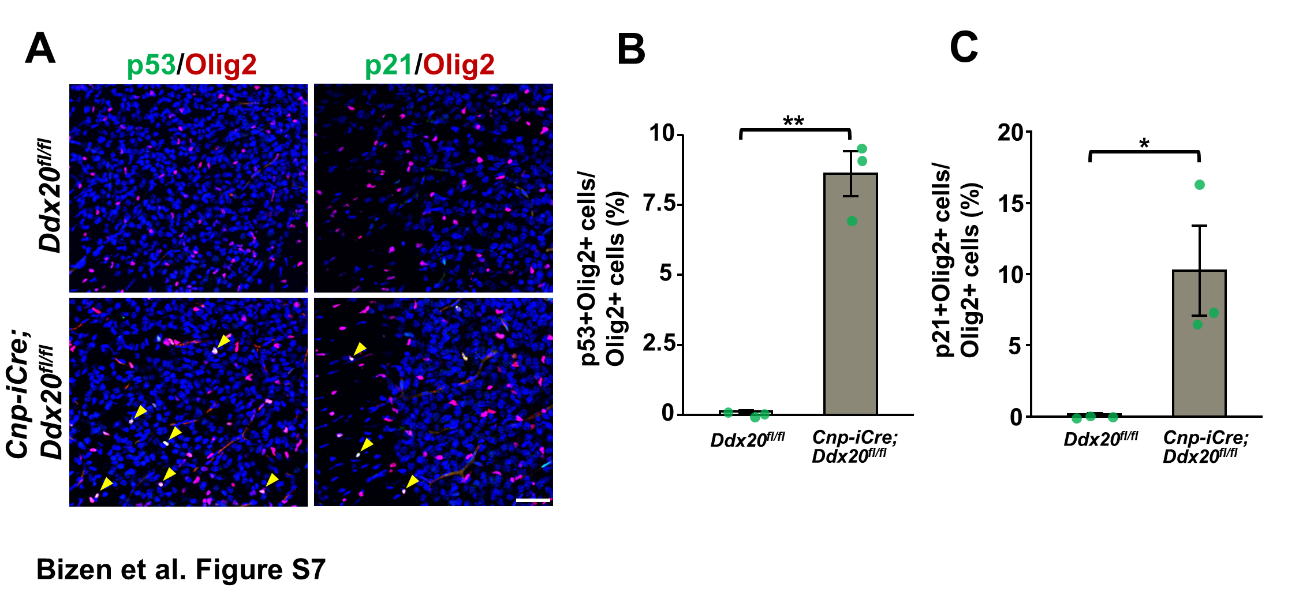
**

**Figure S7. The activation of p53 pathway in OPC-specific *Ddx20* deficient spinal cords.**

(**A**) Immunohistochemistry for p53, p21, and Olig2, in control and *Cnp-iCre;Ddx20* cKO mice. Yellow arrowheads indicate cells that are double-positive for Olig2 and each marker. Scale bars, 50 μm. (**B and C**) The percentage of p53- or p21-positive cells among Olig2-positive cells, shown in (A), respectively. *n* = 3 mice per group. Bar charts represent the mean ± SD. Statistical analysis was performed by two-tailed, unpaired *t*-test. *, *p* < 0.05; **, *p* < 0.01.

**
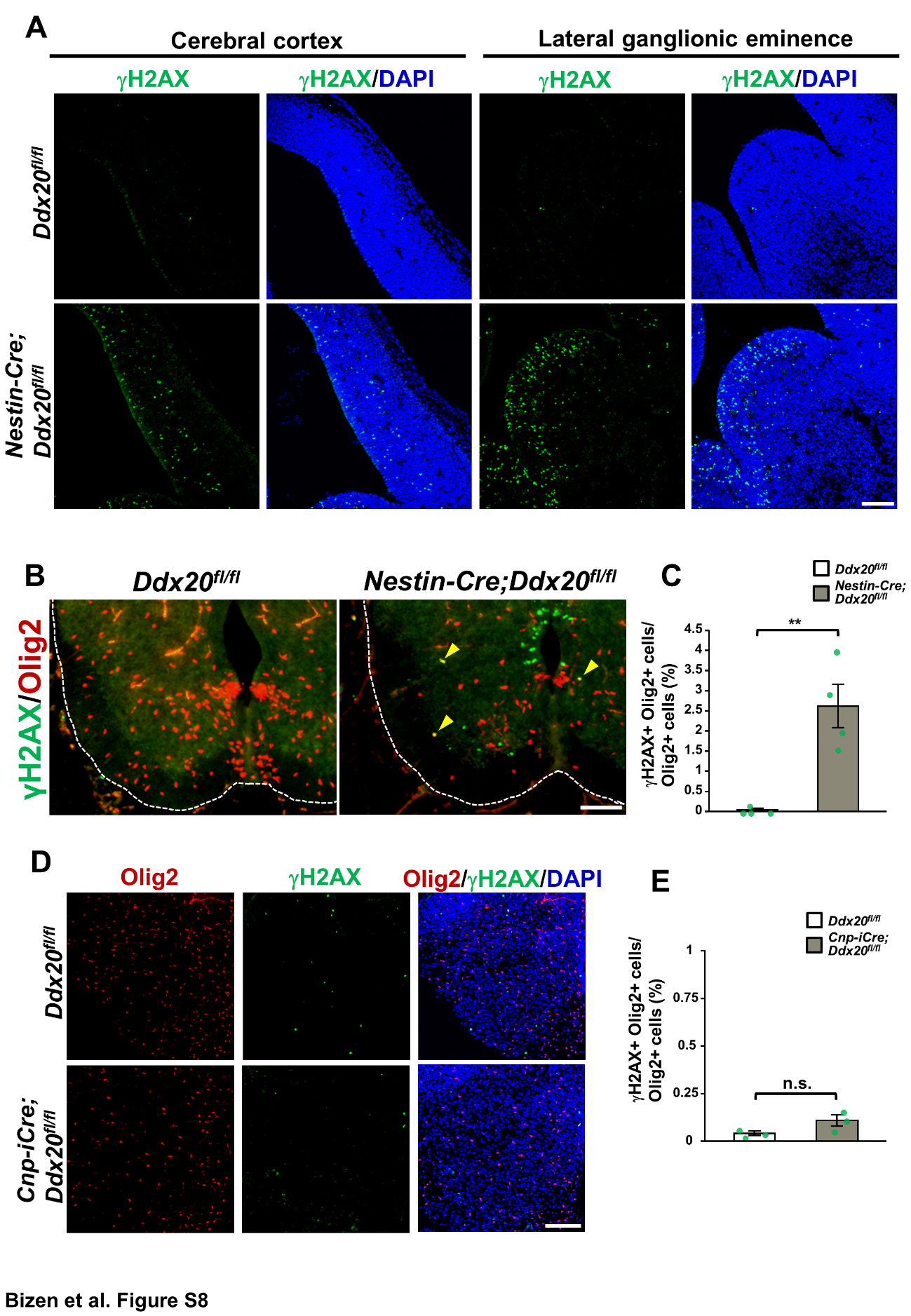
**

**Figure S8. CNS-specific ablation of *Ddx20* induces DNA damage in NPCs and OPCs.**

(**A**) Confocal images of the cerebral cortex and lateral ganglionic eminence of control and *Nestin-Cre;Ddx20* cKO mice. The cryosections were immunostained for γH2AX and DAPI as counterstaining. (**B**) Immunohistochemistry for γH2AX and Olig2 was performed in spinal cords of control and *Nestin-Cre;Ddx20* cKO mice. Yellow arrowheads indicate the cells double-positive for γH2AX and Olig2. (**C**) The bar chart showing the ratio of the cells double-positive for γH2AX and Olig2 to Olig2-positive cells in (B). (**D**) Immunohistochemistry for γH2AX and Olig2 was performed in spinal cords of control and *Cnp-iCre;Ddx20* cKO mice. (**E**) The bar chart showing the ratio of the cells double-positive for γH2AX and Olig2 to Olig2-positive cells in (D). *n* = 4 (C) or *n* = 3 (E) mice per group. Bar charts represent mean ± SD. Statistical analysis was performed by two-tailed unpaired *t*-test. **, *p* < 0.01; n.s., not significant. Scale bars, 100 µm.

**
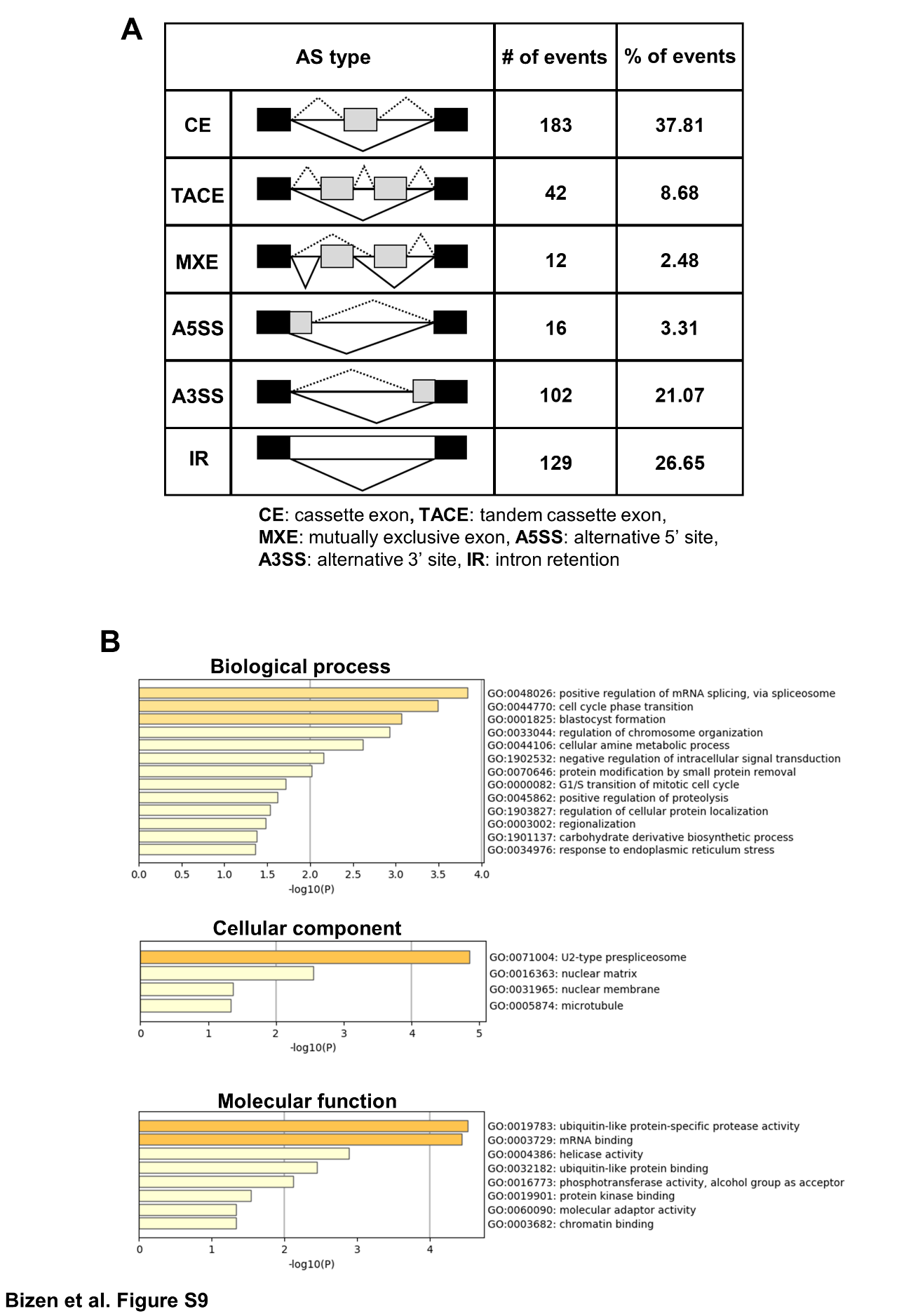
**

**Figure S9. Multiple types of alternative splicing are altered in CNS-specific *Ddx20* cKO spinal cords.** (**A**) Diagram illustrating the patterns of alternative splicing dysregulation in spinal cords of *Nestin-Cre;Ddx20* cKO mice. CE, cassette exon; TACE, tandem cassette exon; MXE, mutually exclusive exon; A5SS, alternative 5' site; A3SS, alternative 3' site; IR, intron retention. Black and gray boxes indicate the remaining exons and skipped exons, respectively, Solid and broken lines indicate the normal and abnormal splicing, respectively. The number or ratio of each event for splicing changes in *Nestin-Cre;Ddx20* cKO spinal cords is shown. (**B**) Gene ontology (GO) analysis using Metascape represents the GO terms (Biological process, Cellular component, Molecular function) of gene group, whose splicing was significantly changed (*p* < 0.001, FDR < 0.1, |Δ*I* | > 0.1) in the *Nestin-Cre;Ddx20* cKO spinal cords.

**
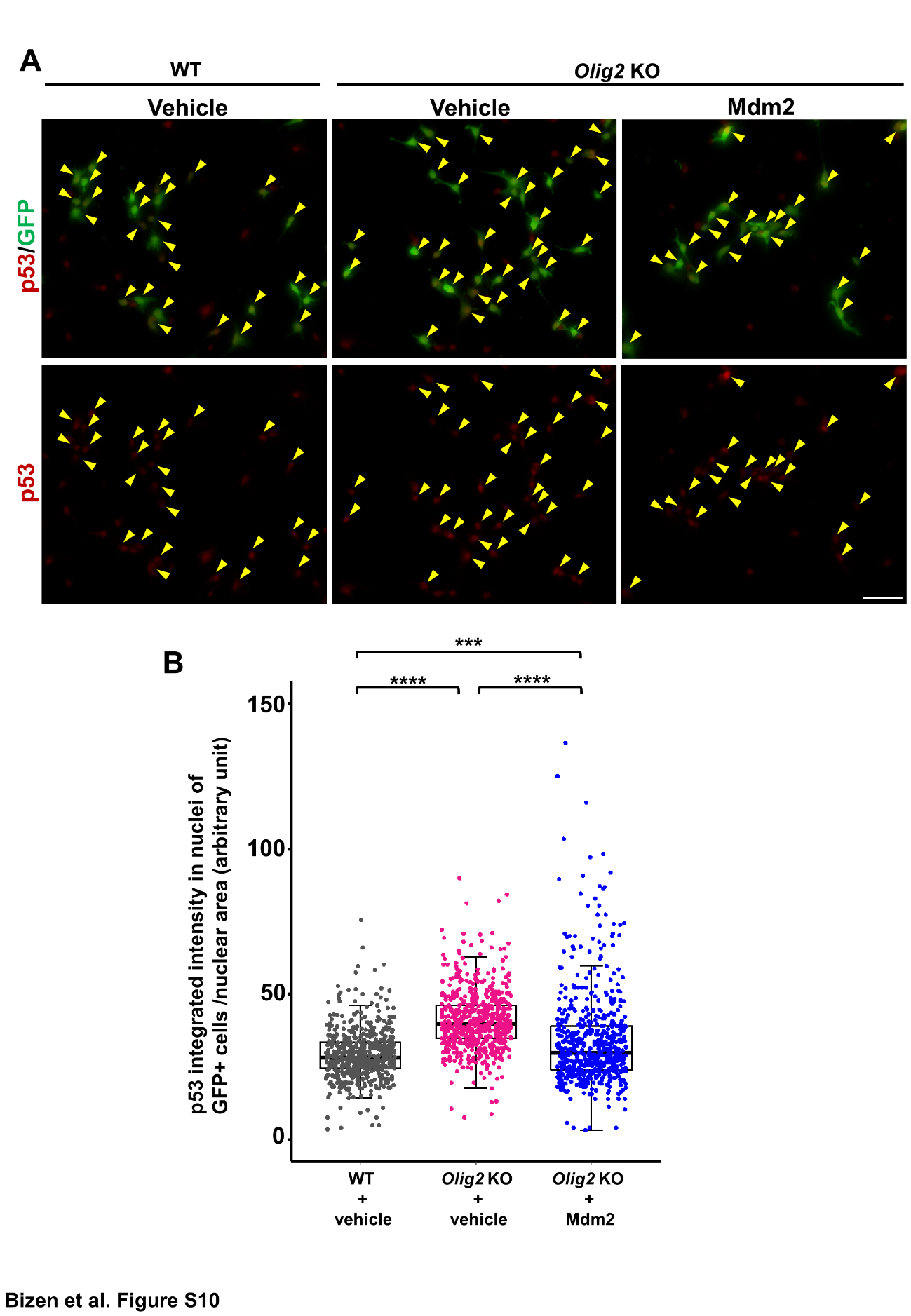
**

**Figure S10. Exogenous expression of exon 3-containing Mdm2 alleviates p53 accumulation in *Olig2* deficient NPCs. A** Double-immunostaining for p53 and GFP, in WT and *Olig2-*KO NPCs, infected with control or *Mdm2*-retrovirus vectors, respectively. Yellow arrowheads indicate cells that are double-positive for p53 and GFP. Scale bar, 50 μm. **B** Box plots (center, median; box, interquartile range; whiskers, 1.5x interquartile range) and dot plots represent the intensity of p53 in the nucleus of each GFP-positive cell. Data were pooled from three biologically independent experiments. At least 150 GFP-positive cells, in each condition, from each experiment, were analyzed. WT+vehicle (gray), *n* = 588; *Olig2*-KO+vehicle (pink), *n* = 617; *Olig2*-KO+Mdm2 (blue), *n* = 606. Statistical analysis was performed by Kruskal-Wallis test, with a post hoc Steel-Dwass test. ***, *p* < 0.001; ****, *p* < 0.0001.

**Supplemental Materials and Methods**

**Yeast Two-Hybrid Screening**

Yeast two-hybrid screening was performed by Hybrigenics Services, S.A.S., Evry, France (http://www.hybrigenics-services.com). The coding sequence for full-length mouse Olig2 (AB038697.1) was PCR-amplified and cloned into pB27 as a C-terminal fusion to LexA (LexA-Olig2). The construct was checked by sequencing the entire insert and used as a bait to screen a random-primed mouse embryo brain (E10.5 and E12.5) cDNA library constructed into pP6. pB27 and pP6 derive from the original pBTM116 and pGADGH plasmids, respectively. 111 million clones (15-fold the complexity of the library) were screened using a mating approach with YHGX13 (Y187 ade2-101::loxP-kanMX-loxP, matα) and L40ΔGal4 (matα) yeast strains as previously described (Fromont-Racine et al., 1997). Fifty-seven His+ colonies were selected on a medium lacking tryptophan, leucine, and histidine, and supplemented with 5 mM 3-aminotriazole to handle bait autoactivation. The prey fragments of the positive clones were amplified by PCR and sequenced at their 5' and 3' junctions. The resulting sequences were used to identify the corresponding interacting proteins in the GenBank database (NCBI) using a fully automated procedure. A confidence score (PBS, for Predicted Biological Score) was attributed to each interaction as previously described (Formstecher et al., 2005).

**Generation of *Ddx20^flox^* mice**

Mice carrying floxed *Ddx20* alleles were generated as follows. Briefly, we constructed a gene-targeting vector containing loxP sites flanking exon 2 of the *Ddx20* gene and an FRT flanked neomycin selection cassette (Supplementary Fig. S1). The targeting plasmid linearized by restriction enzyme was electroporated into RENKA embryonic stem cells (Mishina et al., 2007) in a C57BL/6N background. After selection using neomycin, embryonic stem cell clones with homologous recombination at the targeting site were injected into ICR (RRID: MGI:5462094, SLC Japan) blastocysts and implanted into pseudopregnant females. The male chimeras were then crossed with female C57BL/6N mice (RRID: MGI:5657107, Charles River Japan), and offspring with successful germline transmission of the targeted allele were bred with *Flp* driver mouse lines (Tg(EEF1A1-FLP)66Mim, MGI:2651460) (Takeuchi et al., 2005) for the deletion of neomycin selection cassette, resulting in the generation of heterozygous *Ddx20^flox/+^* mice. The heterozygous mice were subsequently intercrossed to generate homozygous *Ddx20^flox/flox^* mice. Genotyping PCR consisted of 30 cycles of denaturation at 94 ˚C for 30 sec, annealing at 60˚C for 30 sec, and extension at 68˚C for 30 sec. PCR primers were listed in Supplemental Table1.

**Histological analysis and Immunohistochemistry**

Mouse whole heads or whole embryos were fixed with 4% paraformaldehyde (PFA) in 0.1M phosphate buffer (pH7.4) at 4°C overnight and then incubated with 20% sucrose in phosphate-buffered saline (PBS) at 4°C overnight, followed by embedding in OCT compound (Sakura FineTek). Cryosections of 18 µm thickness were prepared by cryostat (CM1850, Leica Biosystems) and mounted on slide glasses. For histological analysis, the frozen sections were stained with hematoxylin and eosin (H&E). For immunohistochemistry, the sections were incubated with PBS for 5 min and treated with antigen-retrieval solution (10 mM citric acid) at 100°C for 5 min. After cooling to room temperature (RT), the sections were incubated for 1 h at RT with PBST (0.1% TritonX-100 in PBS) containing 10% goat serum for blocking and permeabilization. The specimens were further incubated with primary antibodies in PBST containing 10% goat serum (antibody solution) at 4°C overnight. On the following day, after washing three times with PBST, the sections were treated with antibody solution containing fluorescent secondary antibodies at RT for 1 h. The sections were washed three times with PBST and then treated with 4′, 6-diamidino-2-phenylindole (DAPI, 1 µg/ml, Dojindo) for counterstaining. After washing three times with PBS, the sections were mounted in a mounting solution. The images were collected using a confocal laser microscope (FV1200, Olympus), and some images were taken by Olympus microscope (BX53, Olympus) and digital camera system (DP74, Olympus). To obtain the quantitative data, the marker-positive cells were counted on at least three sections from each embryo.

**Co-immunoprecipitation (Co-IP) and western blotting**

The combinations of pCAGMCS2-mouse Olig2-FLAG, pCAGRB-HA-mouse Ddx20, and pCAGRB-HA-mouse Ddx20 were transfected into HEK293 cells (ATCC, #CRL-1573) using Lipofectamine 3000 (Thermo Fisher Scientific) according to the manufacturer’s protocol. Mouse Ddx20 clone was obtained from Genescript. Forty-eight hours after transfection the cells were lysed in IP lysis buffer [50 mM Tris-HCl (pH 7.5), 150 mM NaCl, 1% Nonidet P-40, 0.5% sodium deoxycholate, 1× protease inhibitor mix (Roche), and 1× phosphate inhibitor mix (Roche)] and then incubated with gentle rotation for 30 min at 4°C. The lysates were centrifuged at 15,000 rpm for 15 min at 4°C, and then the supernatants were incubated with mouse monoclonal anti-FLAG M2 antibody (5 µg, Sigma-Aldrich, Cat#F1804) with gentle rotation at 4°C overnight. The samples were treated with 35 µl of 50% slurry Dynabeads protein G (Thermo Fisher Scientific) with gentle rotation for 1 h at 4°C. The immune complexes were washed with lysis buffer twice, high salt washing buffer [50 mM Tris-HCl (pH 7.5), 500 mM NaCl, 0.1% Nonidet P-40, 0.05% sodium deoxycholate] twice and low salt washing buffer [50 mM Tris-HCl (pH 7.5), 0.1% Nonidet P-40, 0.05% sodium deoxycholate]. The immune complexes were boiled in sodium dodecyl sulfate (SDS) sample buffer [2% SDS, 50mM Tris-HCl (pH 6.8), 10% glycerol, 6% β-mercaptoethanol, 0.01% bromophenol blue] at 95°C for 5 min. The denatured samples were separated by electrophoresis in Super Sep Ace gels (Wako) and transferred to Hibond-P PVDF 0.45 (GE Healthcare). The membranes were treated with TBS-T [(25 mM Tris-HCl, 137 mM NaCl, 2.7 mM KCl, 0.05% Tween 20), finally adjusted to pH7.5] containing 5% skim milk (Wako) and incubated with followed by appropriate primary antibodies at 4°C overnight. Following washing using TBST, the membranes were further treated with secondary antibodies for 1 h at RT. For signal detection, Western Lightning Plus-ECL (Perkin Elmer) or ImmunoStar LD (Wako) was used as chemiluminescence substrates. Images were acquired using C-DiGit Blot Scanner (LI-COR Biosciences). For the detection of endogenous interaction between Ddx20 and Olig2, the cultured NPCs were lysed in IP lysis buffer. Following centrifugation, the supernatants were treated with rabbit anti-Ddx20 antibody (10 µg, homemade, Immunogen is 722-740 aa of mouse Ddx20) or normal rabbit IgG (10 µg, MBL, Cat#PM035) at 4°C overnight. The subsequent processes were performed as described above. For the detection of Ddx20, SMN, and Gemin2/6 in mouse CNS, E13.5 mouse whole brains or spinal cords were lysed in radio-immunoprecipitation assay buffer [RIPA; 50 mM Tris-HCl (pH 7.5), 150 mM NaCl, 1% Nonidet P-40, 0.5% sodium deoxycholate, 0.1% SDS, 1× protease inhibitor mix, and 1× phosphate inhibitor mix]. The subsequent processes were performed as described above. In Fig.6, A and B, the intensity of each lane was measured by ImageJ software (https://imagej.nih.gov/ij/).

***In situ* hybridization**

The sections were fixed in 4% PFA for 20 min. Following washing twice in PBS, the sections were treated with 1 μg/mL proteinase K in Tris‐based buffer [50 mM Tris‐HCl (pH 7.6), 5 mM EDTA] for 5 min, and then rinsed in PBS. After fixation in 4% PFA and acetylation in 0.1 m triethanolamine (pH 8.0) and 0.25% acetic anhydride for 10 min, the sections were prehybridized at 65°C for 2–4 h with a hybridization solution containing 50% formamide and saline sodium citrate (SSC) (0.15 M NaCl and 0.015 M sodium citrate in diethylpyrocarbonate-treated water (0.2 mg/mL yeast tRNA, 0.1 mg/mL heparin, 1× Denhardt's solution, 0.2% Tween 20, 0.1% CHAPS, and 5 mM EDTA). The sections were then incubated with a hybridization solution containing diluted digoxigenin-labeled RNA probe at 65°C overnight. The hybridized sections were washed three times with 1× SSC and 50% formamide at 65°C for 30 min twice, and with 0.1× SSC at 65°C for 30 min. The sections were washed twice for 30 min in maleic acid buffer [0.1 M maleic acid (pH 7.5), 0.15 mM NaCl and 0.1% Tween 20] at RT and incubated with alkaline phosphatase‐conjugated sheep anti-digoxigenin antibody (1:2,000, Roche Diagnostics, Cat#11093274910) overnight at 4°C. They were then washed three times in maleic acid buffer for 30 min each and then treated with the color development solution [50 μg/mL 4-nitro blue tetrazolium chloride and 175 μg/mL 5-bromo-4-chloro-3-indolyl-phosphate (Roche Diagnostics)] in alkaline phosphatase buffer [0.1M Tris-HCl (pH 9.5), 0.05M MgCl_2_, 0.1M NaCl and 0.1% Tween 20] for 3–10 h in the dark (59). The following antisense RNA probes specific to mouse genes were used: *Ddx20* (GenBank accession number NM_017397.3, nt 145–2622), *Pdgfrα* (ESTclone, AI098416, Invitrogen), *Olig2*, *Olig1, Cnp* (Takebayashi et al., 2000), *Gfap* (GenBank accession number NM_ 010277, nt 84-1376), *Aldh1l1* (GenBank accession number NM_ 027406, nt 202-2910), *Glast* (GenBank accession number NM_ 148938, nt 567-2198)

**Cycloheximide chase assay**

HA-tagged Ddx20 expression plasmids were cotransfected with or without Myc-Olig2 expression plasmids into Plat-E cells. The cells were treated with cycloheximide (CHX; 200 μg/ml, Wako) for 6, 18, and 24 hours. For endogenous Ddx20 degradation in Olig2 KO NPCs, WT or Olig2 KO NPCs were treated with CHX (100μg/ml) for 4, 8, and 16 hours. The protein stability of Ddx20 was examined by Western blotting. The densities of each protein band were measured using ImageJ. Primary antibodies were used bellow: mouse anti-HA (1:1000, Wako, Cat#01421881); mouse anti-Myc (1:1000, DSHB, 9E10); rabbit anti-Olig2 (1:1000, IBL, Cat#18953); rabbit anti-Ddx20 (1:1000, homemade); mouse anti-β-actin (1:2000, Sigma-Aldrich, Cat#AC-15).

**Retrovirus preparation**

Plat-E cells (Morita et al., 2000) were plated on 10 cm dishes in DMEM with 10% fetal bovine serum. HA-tagged mouse *Ddx20* cDNA or FLAG-Myc-tagged mouse *Mdm2* cDNA was inserted into pMY-IRES-GFP (Morita et al., 2000), and the plasmids were transfected into Plat-E cells using Polyethylenimine (PEI) Max (Polysciences) according to the manufacturer’s protocol. Twenty-four hours after transfection, the medium was exchanged for fresh N2-supplemented DMEM/F-12 with FGF2 (10 ng/mL). After further 24 hours, culture medium containing retroviruses was centrifuged at 6,000 × g for 16–20 hours, and then the retrovirus pellets were resuspended in fresh N2-supplemented DMEM/F-12 containing FGF2. All the recombinant DNA experiments in this manuscript followed the guidelines by Niigata University and Ministry of Education Culture, Sports, Science and Technology of Japan.

**Reverse transcription PCR (RT-PCR) and RT-quantitative PCR (RT-qPCR)**

Total RNA was extracted from mouse embryonic brains, spinal cords, or cultured NPCs using miRNeasy Mini Kit (Qiagen). One µg total RNA was reverse transcribed by Superscript III First-Strand Synthesis System (Thermo Fisher Scientific) according to the recommended manufacturer’s protocol. The PCR for detecting *Mdm2* splicing was performed with PCR Thermal Cycler Dice (TaKaRa) as the following condition: 30 (*Mdm2*) cycles of denaturation at 94˚C for 20 sec, annealing at 62˚C for 20 sec, and extension at 72˚C for 30 sec. The RT-qPCR was performed using StepOnePlus real-time PCR detection system (Applied Biosystems), and the results were obtained by the ΔΔCT method. Mouse *Actb*, *Gapdh,* or 5S rRNA were used as internal controls.

**NPC culture and neurosphere assay**

NPCs were isolated from embryonic day 14.5 mouse ganglionic eminence. The cells were seeded on dishes that have been precoated with poly-L-ornithine and fibronectin, and then cultured for 4 days in N2 (25 µg/ml insulin, 100 µg/ml apo-transferrin, 20 nM progesterone, 100 µM putrescine, 30 nM sodium selenite, 1.27 mg/ml NaHCO_3_)-supplemented Dulbecco’s modified Eagle medium/F-12 [DMEM/F12 (pH7.2), Invitrogen] containing recombinant human FGF2 (10 ng/ml, Pepro Tech). The cells were re-plated on 100 mm dishes for co-immunoprecipitation and western blotting. Otherwise, the cells were re-seeded on 60 mm low adhesive dishes for neurosphere formation and cultured with FGF2 (10 ng/ml) and recombinant murine EGF (10 ng/ml, Pepro Tech) for 7 days.

**Supplementary References**

Formstecher E, Aresta S, Collura V, Hamburger A, Meil A, Trehin A, Reverdy C, Betin V, Maire S, Brun C. et al. 2005. Protein interaction mapping: a Drosophila case study. *Genome Res.* **15**: 376–384

Fromont-Racine M, Rain JC, Legrain P. 1997. Toward a functional analysis of the yeast genome through exhaustive two-hybrid screens. *Nat. Genet.* **16**: 277–282

Mishina M, Sakimura K. 2007. Conditional gene targeting on the pure C57BL/6 genetic background. *Neurosci. Res.* **58**: 105–112

Morita S, Kojima T, Kitamura T. 2000. Plat-E: an efficient and stable system for transient packaging of retroviruses. *Gene Ther.* **7**: 1063–1066

Takebayashi H, Yoshida S, Sugimori M, Kosako H, Kominami R, Nakafuku M, Nabeshima Y. 2000. Dynamic expression of basic helix-loop-helix Olig family members: implication of Olig2 in neuron and oligodendrocyte differentiation and identification of a new member, Olig3. *Mech. Dev.* **99**: 143–148

Takeuchi T, Miyazaki T, Watanabe M, Mori H, Sakimura K, Mishina M. 2005. Control of synaptic connection by glutamate receptor delta2 in the adult cerebellum. *J. Neurosci.* **25**: 2146–2156

**Supplementary Tables**

Supplementary Tables are provided as other type files (Excel)

Table S1: Primers and Molpholino list

Table S2: Data quality summary of RNA sequencing

Table S3: Transcripts level changes in *Nestin-Cre; Ddx20* cKO spinal cords

Table S4: Gene ontology terms for significantly decreased gene expression groups in *Nestin-Cre; Ddx20* cKO spinal cords

Table S5: Gene ontology terms for significantly increased gene expression groups in *Nestin-Cre; Ddx20* cKO spinal cords

Table S6: Alternative splicing changes including all AS types in *Nestin-Cre; Ddx20* cKO spinal cords

Table S7: Gene ontology terms for alternative splicing changes in *Nestin-Cre; Ddx20* cKO spinal cords
